# Aureochrome 1a Is Involved in the Photoacclimation of the Diatom *Phaeodactylum tricornutum*

**DOI:** 10.1371/journal.pone.0074451

**Published:** 2013-09-20

**Authors:** Benjamin Schellenberger Costa, Matthias Sachse, Anne Jungandreas, Carolina Rio Bartulos, Ansgar Gruber, Torsten Jakob, Peter G. Kroth, Christian Wilhelm

**Affiliations:** 1 Institut für Biologie, Universität Leipzig, Leipzig, Germany; 2 Fachbereich Biologie, Universität Konstanz, Konstanz, Germany; Mount Allison University, Canada

## Abstract

Aureochromes constitute a family of blue light (BL) receptors which are found exclusively in heterokont algae such as diatoms (Bacillariophyceae) and yellow-green algae (Xanthophyceae). Previous studies on the diatom *Phaeodactylum tricornutum* indicate that the formation of a high light acclimated phenotype is mediated by the absorption of BL and that aureochromes might play an important role in this process. *P. tricornutum* possesses four genes encoding aureochromes. In this study we confirm the nuclear localisation of the *Pt*AUREO1a, 1b and 2 proteins. Furthermore we studied the physiology of light quality acclimation in genetically transformed *P. tricornutum* cell lines with reduced expression of the aureochrome 1a gene. The results demonstrate that the AUREO1a protein has a distinct function in light acclimation. However, rather unexpectedly AUREO1a seems to repress high light acclimation which resulted in a state of ‘hyper’ high light acclimation in *aureo1a* silenced strains. This was indicated by characteristic changes of several photosynthetic parameters, including increased maximum photosynthesis rates, decreased chlorophyll *a* contents per cell and increased values of non-photochemical quenching in AUREO1a silenced strains compared to wild type cultures. Strikingly, AUREO1a silenced strains exhibited phenotypic differences compared to wild type cells during cultivation under BL as well as under red light (RL) conditions. Therefore, AUREO1a might influence the RL signalling process, suggesting an interaction of AUREO1a with RL perception pathways.

## Introduction

Diatoms are unicellular microalgae which contribute significantly to the global carbon, nitrogen, phosphorus and silica cycles [1], [2], [3]. Although present in nearly all aquatic habitats, diatoms are particularly abundant in cold climates and tend to dominate turbulent and nutrient rich ocean waters. In its natural habitat, phytoplankton is exposed to large variations of light intensity [4] and light quality [5], [6]. Hence, the photoprotective capacity of phytoplankton cells is believed to be an important functional trait of microalgal ecology in the aquatic environment [7]. Diatoms as a phytoplankton group show an extraordinary high capacity to dissipate excessively absorbed light energy safely as heat by non-photochemical quenching (NPQ) [8], [9] and the evolutionary success of diatoms is thought to be closely linked to their ability to cope with these dynamic light conditions [10], [11]. In diatoms, the extent of NPQ is closely correlated to the activity of the xanthophyll cycle (XC) and thus determined by the concentration of the XC pigment diatoxanthin (Dtx) [12].

Considerable progress was made in diatom molecular biology since the development of genetic transformation techniques for diatoms [13] and the sequencing of the genomes of *Thalassiosira pseudonana* and *Phaeodactylum tricornutum* [14], [15]. Still, the molecular basis of light perception in diatoms remains enigmatic [10]. In contrast, the understanding of photoacclimation and its underlying molecular mechanisms is far more comprehensive in higher plants and in green algae. The reduction state of the plastoquinone pool as well as the reduction states of the thioredoxin system and other stromal redox pools are thought to be the major regulators of photoacclimation in the green lineage [16], [17], [18], . The signal transduction of these processes is modulated by several other systems, which perceive for example the evolution of reactive oxygen species, the ATP to ADP ratio or the extend of the proton gradient across the thylakoid membrane [20]. Interestingly, to current knowledge photoreceptors are assumed to be of minor importance for the photoacclimation of green algae and higher plants [16], [21]. In contrast, in diatoms photoreceptors may play a more important role for photoacclimation. Coesel et al. [22] characterised cryptochrome *Pt*CPF1 overexpression lines of *P. tricornutum*, which exhibited altered transcription levels of several photoacclimation associated genes involved in carotenoid and chlorophyll biosynthesis and in photoprotection.

Three families of photoreceptors have been identified in diatoms, the red light (RL) absorbing phytochromes as well as the blue light (BL) absorbing cryptochromes and a recently discovered family of BL photoreceptors named aureochromes [10], [23], [24]. Phytochromes and cryptochromes are widely distributed within eukaryotes, whereas aureochromes are restricted to the stramenopiles [25]. Aureochromes possess an N-terminal DNA binding basic zipper (bZIP) domain and a flavin containing C-terminal LOV (light, oxygen, voltage) domain [23]. Heterologous expression of two aureochromes (*Vf*AUREO1/*Vf*AUREO2) of the multicellular xanthophyte *Vaucheria frigida* as GFP fusion proteins in onion epidermis revealed partial and absolute nuclear localisation, respectively. This, together with the presence of a bZIP domain, supported the notion that aureochromes might represent light regulated transcription factors [23], [26]. Furthermore, knockdown-experiments revealed that *Vf*AUREO1 and *Vf*AUREO2 are involved in the induction of branching and the development of the branch primordials into sexual organs, respectively [23]. However, the biological function of aureochromes in unicellular stramenopiles such as diatoms is still unknown. A recent analysis of the *P. tricornutum* AUREO1a LOV and LOV-Jα domains demonstrated the BL-dependent dimerisation of the LOV-Jα domain [26], which is a prerequisite for bZIP-dependent DNA binding. Furthermore, it was shown that AUREO1a is involved in transcriptional regulation of the cell cycle protein dsCYC2 in *P. tricornutum* and facilitates the transition of the G1 checkpoint of the cell cycle [27]. These data indicate that aureochromes are acting as transcription factors and are involved in the regulation of mitosis in unicellular stramenopiles and in the regulation of photomorphogenesis in multicellular stramenopiles. In *P. tricornutum* four different genes encoding aureochromes have been identified [10].

In a previous study we have shown that photoreceptors are involved in the processes of photoacclimation and photoprotection in diatoms [28]. Cultivation of *P. tricornutum* under low irradiance of BL induced the generation of a high light-adapted phenotype whereas a low light-adapted phenotype was observed for cultures grown under equivalent amounts of red light (RL). The high light-adapted phenotype was characterised by increased maximum photosynthesis rates and an enhanced photoprotective potential. The latter was concluded from an increased NPQ capacity, a larger pool of XC pigments and a higher de-epoxidation state of XC pigments after excess illumination in cultures grown under BL conditions in comparison to cultures grown under RL conditions. These results indicated that the acclimation to high irradiance relies on a BL-mediated photoacclimation in *P. tricornutum*.

It was further shown that under BL conditions several thylakoid membrane proteins were up-regulated compared to RL conditions. Interestingly, the promoter regions of the respective genes exhibited a comparatively high frequency of potential aureochrome binding motives (as inferred from *Vf*AUREO1) whereas no such motives were found upstream of genes which were up-regulated under RL conditions [28]. It was speculated that a blue light activated form of an aureochrome of *P. tricornutum* would act as an inducer or enhancer of high light photoacclimation. Consequently, aureochrome silenced strains should exhibit a reduced high light photoacclimation under BL and WL conditions and should perform similarly as wild type (WT) cells grown under RL conditions.

The aims of the present study were to test this hypothesis and to determine the localisation of aureochromes in *P. tricornutum in vivo*. For this purpose, the intracellular localisation of aureochromes was studied by employing full length protein-GFP fusion proteins of three *P. tricornutum* aureochromes. Furthermore, AUREO1a silencing cell lines were generated and their physiological responses to cultivation under limiting and moderate intensities of BL and RL were investigated. To differentiate between light intensity and light quality driven reactions, the applied experimental design ensured that identical amounts of quanta were absorbed by the cells under BL and RL conditions, respectively.

## Materials and Methods

### Phylogenetic Analysis

The dataset includes 32 currently available aureochrome sequences of stramenopiles from the National Center for Biotechnology Information (NCBI; http://www.ncbi.nlm.nih.gov/) or the Joint Genome Institute (JGI; http://genome.jgi-psf.org/) genome databases. For amino acid sequence alignments the ClustalW web application at GenomeNet (http://www.genome.jp/tools/clustalw/) was used and the default settings for slow/accurate alignment and the output format Phylip were chosen. The alignment was manually refined, yielding 360 amino acid positions (Figure S1). Maximum likelihood analyses by the web application PhyML (http://www.atgc-montpellier.fr/phyml/) were conducted [29]; the substitution model LG [30] was selected with four substitution rate categories. Bootstrap analyses of 100 replicates were performed. Only bootstrap values above 50 are shown in the phylogenetic tree. The resulting tree was imported in the web application FigTree v1.1.2 (http://tree.bio.ed.ac.uk/software/figtree/). Radial tree layout was chosen.

### Cultivation of Algae for Transformation and Screening

The axenic *Phaeodactylum tricornutum* Bohlin (CCAP 3/55; UTEX 646) culture was obtained from the culture collection of algae of the University of Texas at Austin (Austin, Tx, USA). *P. tricornutum* was cultivated in f/2 medium according to Guillard and Lorenzen [31] with half of the original salt content and without added silica. Cells were grown under continuous shaking at 20°C in a 16 h/8 h day/night cycle at 35 µmol photons m^–2^ s^–1^. Solid media contained 1.2% (w/v) Bacto Agar (BD, Sparks, MD, USA) and plated cultures were cultivated under continuous illumination at 75 µmol photons m^–2^ s^–1^.

### RNA Extraction and cDNA Generation

Cells were harvested by centrifugation at 5000 *g* for 5 min. Pellets were frozen in liquid nitrogen and pestled. Powdered cells were treated with RNA extraction reagent (TRIzol® reagent, Life Technologies, Darmstadt, Germany) according to the manufacturer’s instructions. Upon obtaining the aqueous phase the protocol was modified by applying the aqueous phase to a RNA affinity spin column (RNeasy® spin column; Qiagen, Hilden, Germany) in order to minimise contamination with DNA. cDNA was generated according to the manufacturer’s instructions by using a reverse transcription kit (Reverse Transcription System, Promega, Mannheim, Germany).

### Generation of *GFP-AUREO* Fusion Constructs

For the generation of c-terminal GFP fusion proteins full length sequences without stop codon of *P. tricornutum AUREO1a* (49116), *AUREO1aSig* (56684), *AUREO1b* (49458) and *AUREO2* (56688) were amplified from cDNA by blunt end PCR [32] with unmodified primers (Table S1) using a Mastercycler ep gradient (Eppendorf, Hamburg, Germany). Numbers in parentheses correspond to protein IDs of the Joint Genome Institute (JGI) database of *P. tricornutum* v2.0 (http://genome.jgi-psf.org/Phatr2/Phatr2.home.html). The original pPha-T1 *P. tricornutum* transformation vector (GenBank AF219942) [33] was modified to contain a StuI restriction site followed by a GFP sequence as described previously [34]. Furthermore, a slightly modified vector was constructed by mutating the GFP START-codon to GGA. The full length *AUREO* sequences were cloned into both variants of the pPha-T1-GFP transformation vector in frame with GFP. All fusion-constructs were sequenced (GATC Biotech AG, Konstanz, Germany) from their 5′ and 3′ end to verify correct cloning.

### Nuclear Transformation of *P. tricornutum*

Nuclear transformation of *P. tricornutum* was performed using a Bio-Rad Biolistic PDS-1000/He Particle Delivery System (Bio-Rad, Hercules, CA, USA) fitted with 900/1100/1350 psi rupture disks as described previously [13], [35]. For selective cultivation of *P. tricornutum* transformants 75 µg ml^–1^ Zeocin (Invitrogen, Carlsbad, CA, USA) were added to the solid f/2 media according to Guillard and Lorenzen [31].

### Microscopy of Aureochrome:GFP Fusion Expressing *P. tricornutum* cell lines

Cells were observed using an Olympus BX51 epifluorescence microscope equipped with a Zeiss AxioCam MRm digital camera system (Carl Zeiss MicroImaging GmbH, Göttingen, Germany). Differential interference contrast illumination (DIC) was used in order to obtain transmitted light images. Chlorophyll autofluorescence and GFP fluorescence of transformants were dissected using the mirror unit UMWSG2 (Olympus) and the filter set 41020 (Chroma Technology Corp, Rockingham, VT, USA), respectively. Multichannel fluorescence pictures were recorded and assembled with the software AxioVision Rel. 4.6 (Carl Zeiss Microscopy GmbH, Göttingen, Germany). Micrographs were size calibrated using a stage micrometer.

Additionally, images were acquired with a confocal laser scanning microscope LSM 510 META (Carl Zeiss MicroImaging GmbH, Göttingen, Germany) using a Plan-Apochromat 63x/1.4 Oil DIC objective. GFP fluorescence and chlorophyll autofluorescence were excited at 488 nm, Hoechst 33342 DNA stain fluorescence was excited at 405 nm, filtered with a beam splitter (HFT 405/488/543), and detected by three different photomultipliers with a band-pass filter (BP 505–530) for GFP fluorescence, a low pass filter (LP 650) for chlorophyll autofluorescence and a band pass filter (BP 470–500) for Hoechst 33342 DNA stain. For multichannel image acquisition of DIC, GFP fluorescence, chlorophyll autofluorescence and Hoechst 33342 DNA stain fluorescence the software ZEN 2009 was used. For each image z-stacks of 20 pictures were acquired. Maximum intensity z-projections were calculated from slices of image stacks to ensure complete detection of fluorochromes within a cell. Additionally, orthoview analysis of nuclear GFP-co-localisation was performed (Figure S2).

### Generation of an AUREO1a Silencing Construct

For the design of the AUREO1a silencing construct, a 120 bp long unique nucleotide sequence of AUREO1a was identified (bp 982–1101). For verification of the AUREO1a specificity, nucleotide BLAST analyses in the genome of *P. tricornutum* were conducted in the corresponding databases of the JGI and NCBI. The nucleotide sequence was synthesised (Eurofins MWG Operon, Ebersberg, Germany) as part of a synthetic gene construct in the vector backbone pCR2.1 in sense orientation adding three restriction sites: sticky restriction sites XbaI followed by KpnI to the 5′-end and a blunt restriction site HpaI to the 3′-end **(** Figure S3 **)**. The vector was amplified in *Escherichia coli* XL1 Blue (Agilent Technologies, Waldbronn, Germany) and isolated (QIAprep® Spin Miniprep Kit). Mixed sticky and blunt end restriction digestions were performed with KpnI and HpaI in order to obtain the *AUREO1a* fragment for sense orientation and with XbaI and HpaI in order to obtain the *AUREO1a* fragment for antisense orientation.

**Figure 1:**
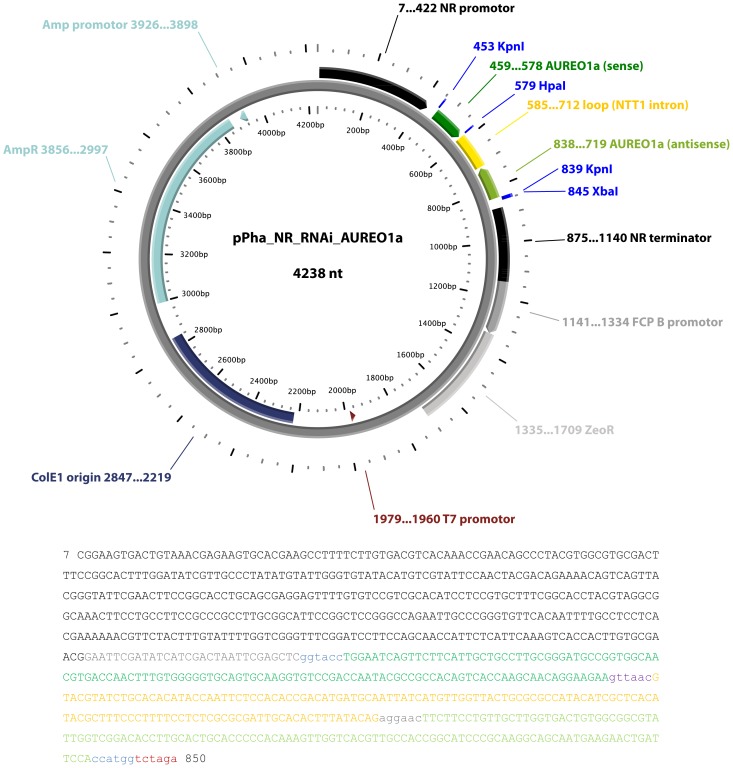
Vector map of the aureochrome 1a silencing construct. The sequence of the NR promoter controlled hairpin construct is given. The sequence is colour coded corresponding to the vector map: NR promoter (black), aureochrome 1a sense (intense green), NTT1 intron (yellow) and aureochrome 1a antisense (light green). Active and inactively fused restriction sites are given in small letters in colour coding: KpnI (light blue), HpaI (purple), inactive fusion of StuI and HpaI (grey) and XbaI (dark red).

The loop region of the RNAi construct consisted of the second intron of the *P. tricornutum* NTT1 gene (49533), which was cloned into the shuttle vector pPha-NR (GenBank accession no. JN180663 [36]; kindly provided by Stefan Zauner/Philipps Universität Marburg). Additional restriction sites were added to the vector (Figure S4). pPha-NR contains the promoter region of the *P. tricornutum* homologue of the nitrate reductase, which was shown to be inducible by nitrate availability in the diatoms *Cylindrotheca fusiformis* and *Thalassiosira pseudonana* [37], [38]. The target vector was cut by a mixed sticky and blunt end restriction digestion with KpnI/HpaI. The sense fragment of *AUREO1a* was inserted upstream of the loop region into the KpnI/HpaI digested target vector. This process was repeated employing XbaI and StuI for the mixed digest of the target vector carrying the sense fragment in order to introduce the second fragment in antisense orientation downstream of the loop region of the target vector to generate the final construct (Figure 1). Constructs were sequenced (GATC Biotech AG, Konstanz, Germany) after each cloning step from 5′ and/or 3′ end of the inserts to ensure correct cloning. Subsequently, nuclear transformation of *P. tricornutum* was performed as described above.

### Screening of AUREO1a Silenced Cell Lines

Small amounts of cell material were collected with sterile toothpicks and suspended in 50 µl distilled water. The samples were subjected to three freeze (−20°C for 30 min) and thaw (room temperature) cycles. Subsequently, samples were incubated at 95°C for 10 min. 1 µl of each sample was taken as template for standard PCR amplification with a total volume of 12.5 µl. Primers annealing in the nitrate reductase promoter of the vector and in the corresponding downstream region of the silencing construct were used (Table S1). PCR products were screened for the occurrence of signals with an amplicon length of 400 bp indicating the insertion of an AUREO1a silencing construct.

Positive clones were cultivated in 50 ml Erlenmeyer flasks in f/2 medium with half of the original salt content and without silica. In addition to growth with NaNO_3_ as sole nitrogen source, each clone was further cultivated in modified f/2 medium with 0.88 mM NH_4_Cl substituting NaNO_3_. Cultures were grown at an incident irradiation of 35 µmol photons m^−2^ s^−1^ white light emitted by fluorescence tubes (18 W/865, Osram, Munich, Germany) and a day/night rhythm of 16 h/8 h until they exhibited a Chl *a* content of approximately 2 µg ml^−1^. Cultures were always harvested at the same time of the day via centrifugation (6 min, 3400 *g*, 4°C). Cell pellets were frozen in liquid nitrogen and stored at −80°C until further use.

### Protein Isolation and Immunoblotting

For protein extraction, cell pellets were resuspended in 100 µl isolation buffer (50 mM Tris HCL, pH 8.0) supplemented with protease inhibitor (Complete™ EDTA-free, Roche, Grenzach Wyhlen, Germany) according to the manufactures instruction. Cells were disrupted by ultrasonification at 4°C. Afterwards, samples were centrifuged for 30 min at 12,000 *g* and 4°C in order to remove cell debris and unbroken cells. The protein concentration of the supernatant was determined via fluorometric protein quantification (Qubit® protein assay kit) according to the manufacturer’s instructions.

Proteins were separated on 12% polyacrylamid gels by one dimensional SDS-PAGE according to Laemmli et al. [39]. 15 µg protein were loaded in each lane if not stated otherwise. Immunoblots were performed as described in Gallagher et al. [40] using an AUREO1a specific peptide antibody (Agrisera AB, Vännäs, Sweden). For the generation of the peptide antibody, the amino acid sequence positions 46–59 of AUREO1a was chosen. The sequence of the synthetic peptide used for immunisation was slightly modified to allow coupling to a carrier protein by adding an N-terminal cysteine: (H_2_N-) CSEQKEELLNSGERE (-CONH_2_). The specificity of the peptide was controlled by a protein BLAST in the JGI database of *P. tricornutum*, which yielded only one non-redundant hit for AUREO1a (settings: Expect: 1.0 E-1; Wordsize: 3; Filter low complexity regions: activated; Perform gapped alignment: deactivated; Scoring Matrix: BLOSUM62). An identical SDS-PAGE was prepared in parallel to serve as loading control and stained with colloidal Coomassie (Roti®Blue, Roth, Karlsruhe, Germany) according to the manufacturer’s instructions allowing the estimation of the protein content per lane and correct loading of proteins. Successful transfer of the protein samples was confirmed by reversible protein staining of the nitrocellulose membrane with Ponceau S (Roth, Karlsruhe, Germany) and by staining the blotted gels with colloidal Coomassie according to the manufacture’s instructions.

### Cultivation of Algae for Physiological Measurements

For physiological measurements, WT *P. tricornutum* and aureochrome 1a silenced strains *aureo1a-15* and *aureo1a-50* were cultivated as described in Schellenberger Costa et al. [28]. Algae were grown semi-continuously in an air-lifted rectangular bioreactor with a depth of 3 cm and a maximal volume of 2.5 l. The temperature was set to 20°C. For cultivation, f/2 medium with half of the original salt content and without silica was used. Algal cultures were maintained at a Chl *a* concentration of about 2 µg ml^−1^. Prior to the measurements, algae were adapted to the specific light conditions for at least one week. Each strain was grown under limiting (LL) and medium light (ML) intensities of blue (469±10 nm; BL) or red light (659±11 nm; RL) at a day/night rhythm of 14 h/10 h. Incident irradiance of BL and RL was carefully adjusted to yield the same amount of photosynthetically absorbed radiation (Q_Phar_) according to Gilbert et al. [41] for all cell suspensions grown under LL and ML conditions, respectively. For LL conditions, cultures were adjusted to a Q_Phar_ of 10 µmol photons m^−2^ s^−1^. For ML conditions, cultures were adjusted to a Q_Phar_ of 30 µmol photons m^−2^ s^−1^. The calculation of Q_Phar_ requires the determination of the Chl *a*-specific *in vivo* absorption coefficient (a*_Phy_) and of the emission spectra of the source of irradiation. The emission spectra of the blue and red LED panels (CLF plants, Wertingen, Germany) used for illumination were measured with a spectroradiometer (Tristan, Hamburg, Germany). Due to differences of the a*_Phy_ between aureochrome 1a silenced strains and WT cells under ML conditions, the amount of applied incident irradiance had to be specifically adjusted for the different strains in order to obtain equal amounts of Q_Phar_ in the semi-continuous cultures which were used for the physiological measurements. Details of the specific intensity of the incident irradiance are presented in Table 1.

**Table 1:** Incident light intensities and cellular parameters.

Parameter	Culture condition		Wildtype	*aureo1a-15*	*aureo1a-50*	*aureo1a-15/*WT	*aureo1a-50/*WT
Incident light intensity	LL	BL	24	24	24		
[mol photons m^−2^ s^−1^]		RL	41	41	41		
	ML	BL	72	60	60		
		RL	124	100	100		
Chl a per cell	LL	BL	0.65±0.04	0.7±0.05	0.51±0.07*	O	−
[pg Chl *a* cell^−1^]		RL	0.56±0.06	0.6±0.04	0.52±0.08	O	O
	ML	BL	0.49±0.02	0.29±0.03*	0.29±0.03*	−	−
		RL	0.46±0.04	0.28±0.03*	0.30±0.03*	−	−
a*_Phy_	LL	BL	9.8±0.7	10.3±0.1	11.3±0.2*	O	+
[m^2^ (g Chl *a*)^−1^]		RL	9.9±0.5	9.8±0.2	11.0±0.5*	O	+
	ML	BL	9.8±0.2	12.0±0.2*	12.2±0.3*	+	+
		RL	9.9±0.3	11.8±0.8*	11.9±0.5*	+	+
Dry weight	LL	BL	22.1±2.2	27.6±1.6*	16.5±2.0*	+	−
[pg cell^−1^]		RL	15.9±0.9	19.5±1.2*	14.1±0.9*	+	−
	ML	BL	18.9±0.9	14.3±1.4*	17.6±1.6	−	O
		RL	18.5±1.7	15.9±1.9*	14.3±1.4*	−	−
Growth rate	LL	BL	0.43±0.04	0.42±0.08	0.57±0.01*	O	+
[µ d^−1^]		RL	0.44±0.12	0.46±0.06	0.50±0.02	O	O
	ML	BL	1.07±0.13	1.00±0.06	0.90±0.25	O	O
		RL	0.74±0.08	0.83±0.05	0.94±0.14*	O	+
1/Φ_C_	LL	BL	14.3±2.4	14.3±0.5	12.5±0.7	O	O
[mol photons (mol C)^−1^]		RL	13.8±0.7	14.7±0.4	15.2±1.3	O	O
	ML	BL	13.4±0.1	14.2±1.2	12.4±0.8	O	O
		RL	20.1±0.5	14.7±1.5*	14.8±1.8*	−	−

*Aureo1a-15* and *aureo1a-50* *P. tricornutum* cultures were grown under illumination with blue (BL) and red light (RL) under limiting light (LL, Q_Phar_ = 10 µmol absorbed photons m^−2^ s^−1^) and medium light (ML, Q_Phar_ = 30 µmol absorbed photons m^−2^ s^−1^) conditions; corresponding WT data of Schellenberger Costa et al. [28] is included as reference. Chl *a* per cell is given in pg cell^−1^, growth rate (µ) in d^−1^, a*_Phy_: Chl *a* specific absorption in m^2^ g Chl *a* ^−1^, dry weight is given in pg cell^−1^ and 1/Φ_C_: quantum requirement in mol absorbed photons mol fixed C^−1^. Mean values are shown with standard deviation (n = 3 for 1/Φ_C_ n = 5 to 9 for other parameters). Mean values of *aureo1a* cultures marked with asterisks (*) are significantly different to the WT culture of the same culturing condition according to one-way ANOVA followed by Holm-Sidak pair wise comparison against WT as control group (p<0.05). O: no significant difference between WT and aureochrome 1a silenced strain; −: significant decrease in aureochrome 1a silenced strain compared to WT; +: significant increase in aureochrome 1a silenced strain compared to WT.

Data on WT cells grown under identical conditions were previously presented and discussed in Schellenberger Costa et al. [28]. The same data serve as reference for the consecutively measured data on the *aureo1a* silenced strains obtained in this study and are included where appropriate for convenient comparison.

### Cellular Parameters

The determination of Chl *a* concentration was performed spectrometrically after extraction with 90% acetone according to Wagner et al. [42] using the equations of Jeffrey and Humphrey [43]. Cell numbers were measured with a particle counter (Z2, Beckman Coulter, Krefeld, Germany). Growth rates were calculated from the daily increase of Chl *a*. These growth rates are identical to those based on daily increase of cell number since the Chl *a* content per cell was constant within each culturing condition. *In vivo* absorption spectra were measured with a scattering corrected spectrophotometer (M500, Zeiss, Jena, Germany) adjusted to a bandwidth of 1 nm. Cellular dry weight was determined according to Su et al. [44]. The carbon-related biomass production (B_C_) and the quantum requirement of carbon fixation (1/Φ_C_) were calculated as described in Su et al. [44].

### Photosynthesis Rates and Non-photochemical Quenching

Oxygen-based photosynthesis rates and fluorescence parameters were measured simultaneously as described in Wagner et al. [42]. Non-photochemical quenching (NPQ) was calculated according to Bilger and Björkman [45].

### Pigment Isolation

For determination of diadinoxanthin (Ddx) concentration, 10 ml of dark adapted culture were harvested on a glass fibre filter, frozen in liquid nitrogen and freeze-dried over night (Labconco FreeZone, ILMVAC GmbH, Illmenau, Germany). Pigment extraction and HPLC analysis were performed as described in Su et al. [44]. For determination of Ddx de-epoxidation after excess light illumination, samples were illuminated with 1000 µmol photons m^−2^ s^−1^ white light for 10 min and frozen in liquid nitrogen prior to freeze-drying. The de-epoxidation state (DES) was calculated as the ratio of diatoxanthin (Dtx) to the sum of both pigments: (Dtx)/(Ddx+Dtx).

### Statistics

Statistical analysis of physiological data was carried out by one-way analysis of variance (ANOVA) followed by a Holm-Sidak pair-wise comparison with the WT as control group using the program Sigma Plot 11.0 and a P-value <0.05 for the rejection of the null hypothesis.

## Results

### Phylogenetic Analysis of Aureochromes

**Figure 2:**
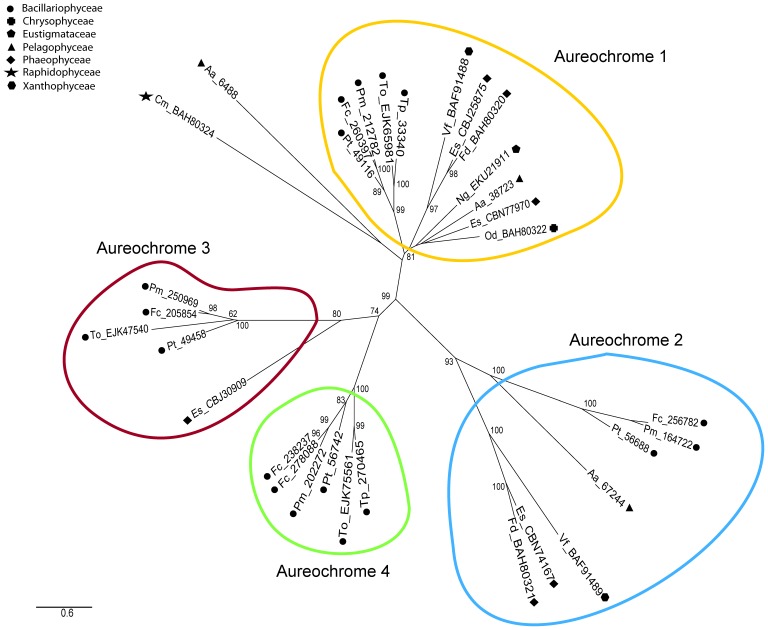
Phylogenetic analysis of putative aureochromes from different stramenopiles. The maximum likelihood tree was calculated using PhyML 3.0 [29] and incorporates 32 putative aureochrome sequences of twelve different stramenopiles identified by the unique bZIP/LOV domain setup. Numbers at nodes of subtrees correspond to bootstrap values greater than 45. The accession numbers correspond either to Protein IDs (only digits) from the Joint Genome Institute database (JGI; http://www.jgi.doe.gov/) or to the accession numbers (two letters and digits) from the database of the National Center for Biotechnology Information (NCBI; http://www.ncbi.nlm.nih.gov/). Putative aureochromes of *Phaeodactylum tricornutum* (Pt), *Fragilariopsis cylindrus CCMP 1102* (Fc), *Pseudo-nitzschia multiseries* (Pm), *Thalassiosira pseudonana* (Tp), *Thalassiosira oceanica* (To), *Ectocarpus siliculosus* (Es), *Fucus distichus subsp. Evanescens* (Fd), *Aureococcus anophagefferens* (Aa), *Chattonella marina var. Antiqua* (Cm), *Ochromonas danica* (Od), *Nannochloropsis gaditana CCMP526* (Ng) and *Vaucheria frigida* (Vf) were taken into account. Four distinct groups of aureochromes could be identified highlighted by coloured markings. The different groups were designated aureochromes 1, 2, 3 and 4 with aureochromes 1 and 2 corresponding in homology to the aureochromes 1 and 2 of *V. frigida* [23], respectively.

In order to classify the four different aureochromes of *P. tricornutum* we performed phylogenetic analyses of aureochrome sequences presently available at NCBI and JGI genome databases. 32 putative aureochromes of twelve different organisms were identified by their conserved bZIP and LOV domain set-up and used for the generation of a maximum-likelihood phylogenetic tree using PhyML analyses (Figure 2). The aureochromes can be divided into four groups, which are characterized by group specific homologies especially between the bZIP and the LOV domain (Figure S1). Groups 1 and 2 are clearly distinct protein families corresponding to the two classes of aureochromes described for *V. frigida* [23]. Groups 3 and 4 are more related to each other but form distinct groups as indicated by a bootstrap value of 74 at the respective junction. Interestingly, in the given data set diatoms are the only organisms that feature group four aureochromes and the majority of group three aureochromes were found in diatoms as well. Remarkably, *P. tricornutum* possesses exactly one aureochrome per group and all investigated diatom genera feature both a class three and a class four aureochrome which further supports the distinction between the different groups. The two aureochromes of *Chattonella marina var. antiqua* and *Aureococcus anophagefferens* could not be assigned to any of the groups.

### Localisation of *P. tricornutum* Aureochromes

**Figure 3:**
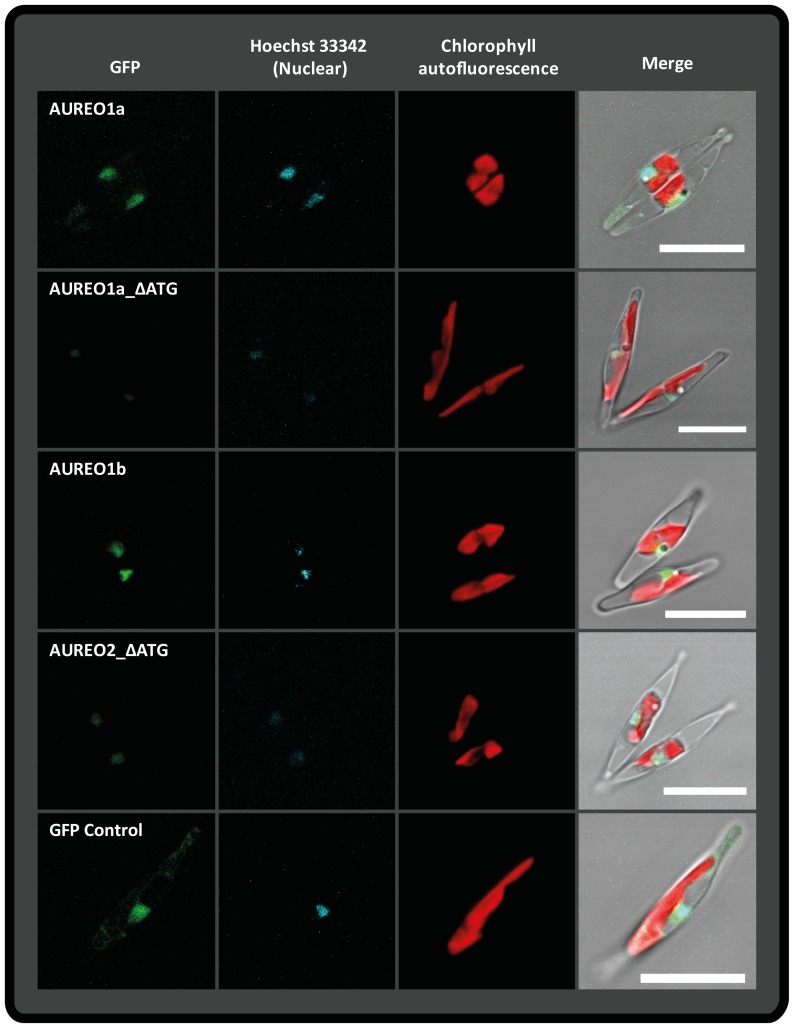
Localisation of GFP-fusion proteins of *P. tricornutum* aureochromes. Maximum intensity z-projections of LSM analyses are shown. From left to right: GFP fluorescence (green), nucleus staining Hoechst 33342 dye (cyan), chlorophyll autofluorescence (red) and a merge of all channels with a representative DIC single plane. The white scale bars correspond to 10 µm. All aureochromes feature a distinct nuclear localisation. AUREO1a fusion proteins often exhibit additional cytosolic signals (Figure S6), as can be seen here for AUREO1a. GFP_control is a transformed cell line of *P. tricornutum* featuring the enhanced GFP protein (GenBank Accession number AAB08060.1), which is missing any targeting sequence. It serves as reference for cytoplasmatic localisation. Here, the z-projections imply a co-localisation in the nucleus as well, but orthoview analysis of the LSM data revealed that the GFP fluorescence was only accumulating around the nucleus, while it co-localises in case of the aureochromes (Figure S2).

As aureochromes are putative transcription factors possessing a bZIP domain, they might influence the physiology of the cells via gene regulation. A prerequisite for a protein to function as a nuclear transcription factor is an, at least temporary, nuclear location. We checked for putative nuclear localisation sequences (NLS) using the program NLStradamus [46] and found a high probability for a single NLS in each of the four *P. tricornutum* aureochromes (Figure S5). Generally, the aureochrome genes found in *P. tricornutum* apparently do not possess N-terminal targeting signals, even in cases of remaining uncertainty about the exact exon/intron boundaries or about the extend of open reading frames (Table S2). The only possible exception is *AUREO1a*. Here, the reading frame of the main gene model can be extended upstream to include a potential signal peptide according to prediction by SignalP 3.0 [47] (Table S2, see protein ID 56684). Hence, we also decided to study the localisation of the potential alternative gene product. We were not able to amplify the cDNA of *AUREO1c* (56742), possibly due to a low expression of this particular gene as indicated by a low number of EST sequences found in the JGI database. Accordingly, we have designed GFP fusion constructs for the EST supported full-length sequences of aureochromes 1a, 1b and 2 and for the alternative version of *AUREO1a* featuring the putative signal peptide (1aSig). To exclude the possibility that the C-terminal GFP domain might be translated via its original ATG start codon, this codon was mutated to GGA and the transformed cell lines (marked by _ΔATG) were analysed as well. After biolistic transformation of wild type *P. tricornutum* using these constructs, we could not detect any difference in the location of AUREO:GFP and the corresponding AUREO:GFP_ΔATG fusion-proteins. Epifluorescence as well as laser scanning microscopy analysis of GFP expression together with Hoechst 33342 DNA staining revealed a clear nuclear location of all three investigated aureochromes (Figure 3 and Figure S2). AUREO1b and AUREO2 were exclusively located in the nucleus (demonstrated by the nuclear stain Hoechst 33342). The AUREO1a fusion proteins showed a dominantly nuclear GFP fluorescence at all times, however, we also detected some weak GFP fluorescence in the cytosol (Figure 3 and Figures S2 and S6). Cell lines successfully transformed with any of the three AUREO1a-GFP fusion constructs exhibited similar phenotypes with strong GFP fluorescence in the nucleus and weak fluorescence in the cytosol (Figure S6). This indicates that the N-terminal extension either does not serve as a signal peptide or that its signal peptide properties are weaker than the nuclear translocation signal.

### Screening of AUREO1a Deficient Cell Lines

**Figure 4:**
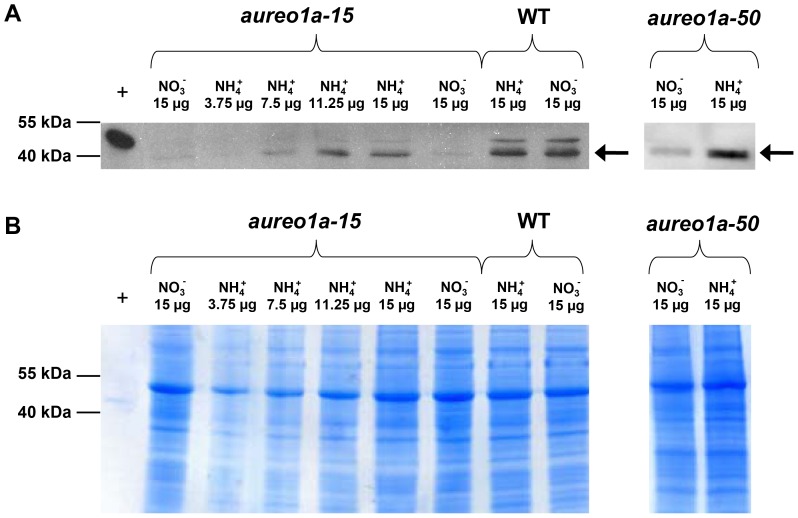
A) Exemplary relative quantification of AUREO1a concentration in protein extracts of *P. tricornutum* WT, *aureo1a-15* and *aureo1a-50* by an immunoblot using an antiserum specific for *P. tricornutum* AUREO1a. Cultures were grown with either nitrate or ammonium as sole nitrogen source. Nitrate activates the promoter of the applied silencing construct resulting in a decreased amount of AUREO1a protein. Several dilutions of the protein extract of the ammonium grown *aureo1a-15* culture were loaded on the gel in order to assess the efficiency of AUREO1a downregulation. Two co-regulated bands are visible, one at the expected size of AUREO1a, 41.5 kDa (indicated by arrows), and a weaker band at about 47 kDa, which possibly reflects a post-translational modification of the protein. **B)** Loading control of protein extracts used for immunoblotting. The gels used for immunoblotting and as loading control were loaded with identical amounts of protein. The proteins of the loading control gels were stained with colloidal Coomassie. +: Purified heterologously expressed AUREO1a with His-tag.

In order to understand the cellular roles of aureochromes in *P. tricornutum*, we investigated aureochrome knock-down cell lines. The most promising aureochrome for this investigation was AUREO1a, because, in contrast to the other aureochromes, it is apparently located both in the nucleus and in the cytoplasm, suggesting a unique functional importance. The other investigated aureochromes are missing similar distinctive features and AUREO1b possesses a less conserved bZIP DNA binding domain (Figure S7). For confirmation of gene silencing, it is mandatory to follow the protein level of the silenced protein and for AUREO1a an antiserum was available. An inducible vector containing an *AUREO1a* gene fragment cloned in sense and antisense orientation to facilitate gene silencing was generated following the method described by Lavaud et al. [48]. Transformed *P. tricornutum* cells were screened for reduced AUREO1a protein content via immunoblotting of three independent replicates using the antiserum specific for AUREO1a. The antiserum labelled two bands, one at 41.5 kDa, the expected size of AUREO1a, and a clearly weaker band at about 47 kDa. The intensities of these two bands relative to each other were identical in all samples that were taken from cells grown under different conditions, indicating that the upper band represents a posttranslationally modified form of AUREO1a. In the strains *aureo1a-15* and *aureo1a-50,* an obvious decrease of AUREO1a protein levels was observed for cultures cultivated with nitrate as sole nitrogen source instead of ammonium (Figure 4A, corresponding loading controls are depicted in Figure 4B). This indicates a successful integration of the aureo1a silencing construct. As the construct is driven by an NR promoter we only observed AUREO1a reduction in nitrate containing media. We furthermore could show that the nitrogen source has no influence on the amount of AUREO1a protein in WT control cells. In both silenced strains, *aureo1a-15* and *aureo1a-50*, AUREO1a levels of cultures grown with nitrate as sole nitrogen source were equally reduced to below 50% of the AUREO1a content of cultures grown with ammonium as sole nitrogen source.

### Cellular Parameters

To determine the role of AUREO1a in the regulation of physiological processes, several cellular parameters of the aureochrome 1a silenced strains *aureo1a-15* and *aureo1a-50* were compared to WT cells. Under low light (LL) conditions, the Chl *a* content per cell of *aureo1a-15* was comparable to that of the corresponding WT culture whereas *aureo1a-50* exhibited a slightly decreased Chl *a* content per cell. Consequently, only *aureo1a-50* cultures showed a slightly increased Chl *a*-specific *in vivo* absorption (a*_Phy_) at LL conditions compared to WT cells (Table 1).

Differences between WT and aureochrome silenced strains were more pronounced at medium light (ML) conditions. Here, the Chl *a* content per cell in both aureochrome 1a silenced strains was decreased to about 60% compared to the corresponding WT cultures grown at both light qualities. As a consequence, a*_Phy_ was increased by approximately 20% in both aureochrome 1a silenced strains under ML conditions (Table 1). The elevated a*_Phy_ of the aureochrome 1a silenced strains were taken into account for the adjustment of equal levels of Q_Phar_ (see Methods section).

The comparison of cellular dry weight between WT cells and mutant strains did not yield in a consistent pattern. Compared to WT cells, the cellular dry weight of *aureo1a-50* was decreased at almost all culture conditions, whereas *aureo1a-15* cultures exhibited an increased cellular dry weight at LL conditions but a decreased cellular dry weight at ML conditions (Table 1).

At all culture conditions, the growth rates of *aureo1a-15* cultures were comparable to those of WT cells (Table 1). Although growth rates were generally higher under ML compared to LL conditions for WT and *aureo1a-15*, this difference was more pronounced under BL (LL: 0.42 d^−1^; ML: 1.00 d^−1^) than under RL conditions (LL: 0.46 d^−1^; ML: 0.83 d^−1^). Compared to the WT cells, cultures of *aureo1a-50* exhibited increased growth rates under LL conditions. Interestingly, under ML conditions similarly increased growth rates were detected for *aureo1a-50* in comparison to WT cells only in combination with RL but not with BL. Thus, only *aureo1a-50* exhibited a clearly different growth performance in comparison to WT cells.

The quantum requirement of carbon fixed in the biomass (1/Φ_C_) is the most integrating growth parameter since it incorporates all energetic losses of the cellular metabolism. Despite the differences of cellular Chl *a* content and cellular dry weight between WT cells and aureochrome 1a silenced strains, 1/Φ_C_ exhibited a comparable pattern to the growth rates. No significant differences between WT cultures and cultures of both aureochrome 1a silenced strains were observed in BL irrespective of LL or ML conditions (Table 1). Under these conditions quantum efficiency varied only slightly between 12.5±0.7 and 15.2±1.3 mol absorbed photons mol per fixed C. Interestingly, the clear increase in quantum requirement of WT cells under ML RL conditions was not observed for both aureochrome 1a silenced strains.

### Photosynthesis Rates

**Figure 5:**
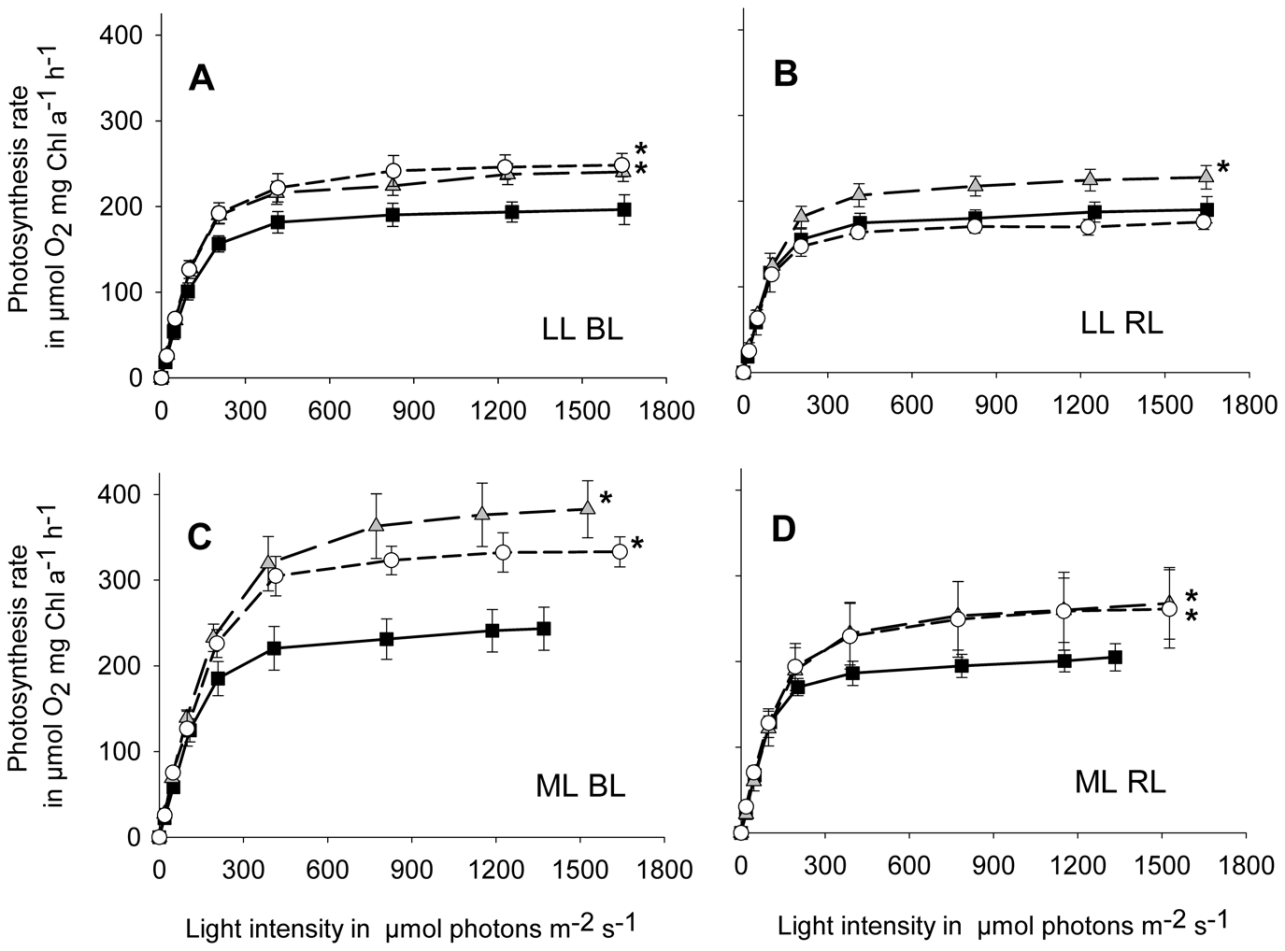
Photosynthesis rates of *aureo1a-15* (grey triangles) and *aureo1a-50* (white circles) *P. tricornutum* cultures depending on the incident light intensity in the measuring cuvette; corresponding WT data of Schellenberger Costa et al. [**28**] **(black squares) are included for better comparison.** Algae were cultivated at a Q_Phar_ of 10 µmol absorbed photons m^−2^ s^−1^ (LL) under illumination with blue (A) and red light (B) and further at a Q_Phar_ of 30 µmol absorbed photons m^−2^ s^−1^ (ML) under illumination with blue (C) and red light (D). Mean values are shown with standard deviation (n = 9). Maximum photosynthesis rates of each culture condition were tested for the occurrence of significant differences between WT and aureochrome 1a silenced strains using a one-way ANOVA followed by a Holms-Sidak pair-wise comparison test with the WT as control group. Significant differences are marked with asterisks (*p*<0.05).

When cultivated at LL BL, both aureochrome 1a silenced strains exhibited significantly increased photosynthesis rates compared to WT cells with maximum photosynthesis rates (P_Max_) of about 240 µmol O_2_ mg Chl a^−1^ h^−1^ (*aureo1a*) compared to 196 µmol O_2_ mg Chl a^−1^ h^−1^ (WT; Figure 5A). At LL RL only *aureo1a-15* exhibited a significantly increased P_Max_ in comparison to WT cultures (Figure 5B).

Cultivation at ML BL resulted in remarkably high photosynthesis rates with a P_Max_ of 382 µmol O_2_ mg Chl a^−1^ h^−1^ for *aureo1a-15* and 332 µmol O_2_ mg Chl a^−1^ h^−1^ for *aureo1a-50*. These rates were significantly higher than the P_Max_ of the corresponding WT culture (243 µmol O_2_ mg Chl a^−1^ h^−1^; Figure 5C). A comparable pattern was observed under ML RL. The P_Max_ values of both aureochrome 1a silenced strains (about 265 µmol O_2_ mg Chl a^−1^ h^−1^) were significantly increased compared to P_Max_ of corresponding WT culture (205 µmol O_2_ mg Chl a^−1^ h^−1^, Figure 5D). Hence, the *aureo1a* cultures exhibited a high light acclimation status clearly exceeding that of WT cultures grown under identical conditions. Interestingly, the trend of increased photosynthesis rates under ML in comparison to LL conditions was observed for both BL and RL aureochrome 1a silenced cultures, albeit more pronounced under BL conditions. In contrast, in WT cells this effect was only detected under BL conditions.

### Non-photochemical Quenching and XC Pigment Pool Size

**Figure 6:**
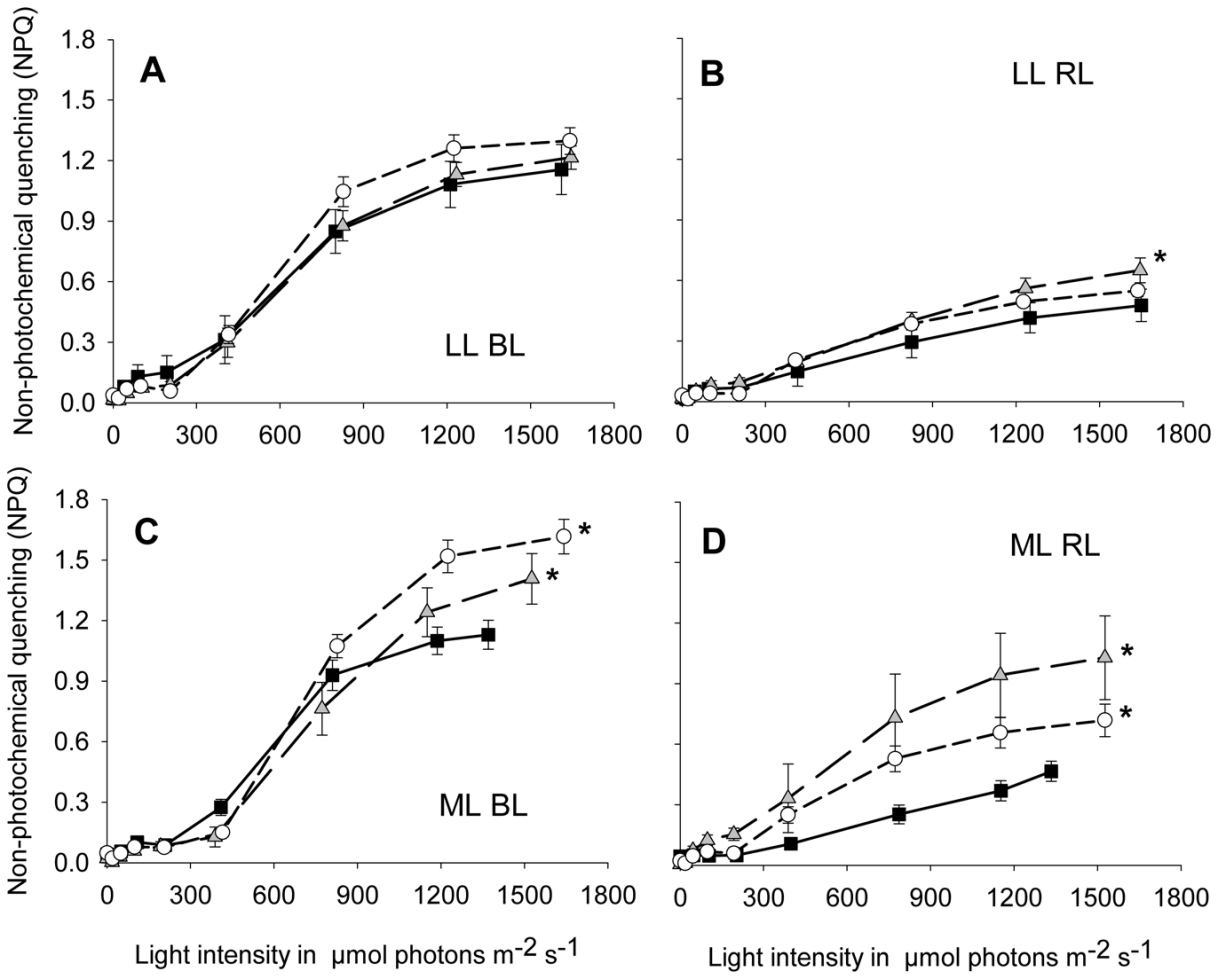
Non-photochemical quenching (NPQ) of *aureo1a-15* (grey triangles) and *aureo1a-50* (white circles) *P. tricornutum* cultures depending on the incident light intensity in the measuring cuvette; corresponding WT data of Schellenberger Costa et al. [**28**] **(black squares) are included for better comparison.** Algae were cultivated at a Q_Phar_ of 10 µmol absorbed photons m^−2^ s^−1^ (LL) under illumination with blue (A) and red light (B) and further at a Q_Phar_ of 30 µmol absorbed photons m^−2^ s^−1^ (ML) under illumination with blue (C) and red light (D). Mean values are shown with standard deviation (n = 9). The maximum NPQ of each culture condition was tested for the occurrence of significant differences between WT and aureochrome 1a silenced using a one-way ANOVA followed by a Holms-Sidak pair-wise comparison test with the WT as control group. Significant differences are marked with asterisks (*p*<0.05).

In parallel to the measurement of light response curves, fluorescence parameters were recorded which, in combination with excess illumination experiments, were used to evaluate the capacity of NPQ and the XC pigment pool size of *aureo1a* strains in comparison to the WT cells. WT cells of *P. tricornutum* show a significantly higher NPQ capacity after cultivation under illumination with BL as compared to illumination with RL, irrespective of LL or ML conditions [28]. Under LL conditions this pattern was also observed in both aureochrome 1a silenced strains with only slightly increased NPQ compared to WT cells (Figure 6A & B). However, under ML conditions significantly increased maximal NPQ values were observed in both aureochrome 1a silenced strains in comparison to WT cells irrespective of the light quality (Figure 6C & D). The highest NPQ values of the *aureo1a* strains at ML were higher than the respective values at LL for both BL and RL cultures (compare Figure 6A & C and Figure 6B & D). In contrast, there was no change in maximum NPQ between LL or ML conditions in the WT cells.

For both aureochrome 1a silenced strains significantly higher diadinoxanthin (Ddx) concentrations were found in comparison to WT cells at almost all growth conditions. It is noteworthy that the Ddx content was usually higher in *aureo1a-15* than in *aureo1a-50* (Table 2). Moreover, in WT cells the Ddx concentration was only increased comparing ML to LL under BL conditions but not under RL conditions. In contrast, *aureo1a* strains exhibited increased Ddx concentrations in all ML conditions compared to the corresponding LL.

**Table 2:** Diadinoxanthin (Ddx) concentration and de-epoxidation state (DES).

Parameter	Culture condition		Wildtype	*aureo1a-15*	*aureo1a-50*	*aureo1a-15/*WT	*aureo1a-50/*WT
Ddx under culture	LL	BL	110±4	125±7*	119±6*	+	+
conditions		RL	82±4	90±4*	89±2*	+	+
[mmol (mol Chl *a*) ^−1^]	ML	BL	130±20	165±13*	145±11	+	O
		RL	83±3	134±22*	106±15*	+	+
DES after illumination	LL	BL	0.38±0.01	0.40±0.03	0.40±0.01	O	O
with excess light		RL	0.26±0.01	0.30±0.01*	0.28±0.01*	+	+
[r.U.]	ML	BL	0.45±0.01	0.43±0.04	0.48±0.02	O	O
		RL	0.29±0.02	0.44±0.03*	0.42±0.05*	+	+

*Aureo1a-15* and *aureo1a-50* *P. tricornutum* cultures were grown under illumination with blue (BL) and red light (RL) under limiting light (LL, Q_Phar_ = 10 µmol absorbed photons m^−2^ s^−1^) and medium light (ML, Q_Phar_ = 10 µmol absorbed photons m^−2^ s^−1^) conditions; corresponding WT data of Schellenberger Costa et al. [28] is included as reference. Ddx-concentration is given in mmol mol Chl *a* ^−1^. DES was measured after 10 min of illumination with 1000 µmol photons m^−2^ s^−1^ as the ratio of diatoxanthin (Dtx) to Ddx+Dtx. Mean values are shown with standard deviation (n = 5 to 9). Mean values of *aureo1a* cultures marked with asterisks (*) are significantly different to the WT culture of the same culturing condition according to one-way ANOVA followed by Holm-Sidak pair wise comparison against WT as control group (p<0.05). O: no significant difference between WT and aureochrome 1a silenced strain; −: significant decrease in aureochrome 1a silenced strain compared to WT; +: significant increase in aureochrome 1a silenced strain compared to WT.

Table 2 presents de-epoxidation states (DES) of XC pigments which were determined after a 10 min period of illumination with 1000 µmol photons m^−2^ s^−1^. The DES is depicted as the ratio of diatoxanthin (Dtx) per (Ddx+Dtx). Generally, an elevated DES was observed in WT cells and *aureo1a* strains under BL conditions compared to RL conditions. No differences of the DES could be detected in WT cells to both aureochrome 1a silenced strains under BL conditions. However, under RL conditions the DES values were always significantly higher in the aureochrome 1a silenced strains. Interestingly, this resulted in the observation that in *aureo1a-15* the DES after excess light illumination was comparable for ML RL and ML BL cultures. The highest DES was measured in *aureo1a-50* cultures cultivated under ML BL. In summary, cultures of aureochrome 1a silenced strains tended to exhibit an increased high light acclimation status compared to the corresponding WT cultures concerning NPQ capacity, Ddx concentration and Ddx de-epoxidation.

## Discussion

High light acclimation in diatoms is typically associated with a decrease of the cellular Chl *a* content and enhanced maximum photosynthesis rates. Furthermore, the photoprotective potential is clearly increased which is usually accompanied by an increased pool size of xanthophyll cycle pigments and an accelerated de-epoxidation of Ddx to Dtx under excess light conditions [28], [49], [50], [51], [52]. High light acclimation is further connected to the up- or down-regulation of the expression of specific genes involved in photoprotection or light harvesting [53], [54], [55], [56] as well as other processes such as the carbon metabolism [54]. Similar to high light acclimation, also the re-acclimation to illumination after prolonged darkness is accompanied by extensive transcriptional changes [57]. In a previous study we have shown that RL is not a trigger for light acclimation in WT cells of *P. tricornutum* and that the formation of an apparently high light acclimated phenotype is mediated by the absorption of BL despite moderate light intensities at cultivation [28]. Based on these results it was suggested that aureochromes might play an important role in the process of BL perception and that the active form of one or more of the aureochromes might act as transcription factors and induce or enhance the acclimation to higher light intensities. The predicted nuclear localisation of all four aureochromes and the confirmation by successful GFP fusion experiments for three of them further support a role of the aureochromes as transcription factors. Interestingly, AUREO1a was detected both in the nucleus and in the cytoplasm, indicating a different functionality compared to the other aureochromes, possibly involving shuttling between cytoplasm and nucleus.

To study the influence of AUREO1a on the photoacclimation of *P. tricornutum*, we cultivated the aureochrome 1a silenced strains at BL and RL of different light intensities. Surprisingly, the results suggest a regulation of BL mediated light acclimation which stands in clear contrast to our expectations. At LL conditions, WT cells and the aureochrome 1a silenced strains showed very similar physiological properties under illumination with both BL and RL. This indicates that AUREO1a is not of major importance for the photoacclimation of *P. tricornutum* at low light intensities. Instead, the observed differences between the BL and RL acclimated phenotypes of the WT might be mediated by other BL receptors like other aureochromes or members of the cryptochrome family.

At ML conditions, the physiological response of AUREO1a silencing cell lines showed explicit characteristics of acclimation to increased light intensities irrespectively of the applied light quality, including a reduction of the cellular Chl *a* content and the cellular dry weight as well as increased photosynthesis rates and an enhanced photoprotective potential. Hence, it can be stated that aureochrome 1a silenced cultures were ‘hyper’ acclimated under ML illumination suggesting that AUREO1a is involved in the photoacclimation of *P. tricornutum*. Considering the lacking influence of AUREO1a on the phenotype of LL cultures, this indicates the presence of a light intensity perception mechanism which is negatively affected by aureochrome 1a and which may comprise the reduction states of stromal compounds or the reduction state of the PQ pool. This would correspond to the recent observation that the nuclear gene expression is influenced by the addition of inhibitors of the linear electron transport in *P. tricornutum* [56].

Unexpectedly, *aureo1a* cultures showed different characteristics compared to WT cells also under ML RL conditions. The physiological parameters of the *aureo1a* cultures clearly showed signs of an acclimation to increased light intensities whereas WT cells showed no corresponding acclimation at ML RL conditions in comparison to LL RL conditions. Hence, in aureochrome 1a silenced strains RL was able to trigger a limited acclimation to increased light intensities. A possible explanation for these results could be that AUREO1a does not induce or enhance high light acclimation but, to the contrary, acts as a repressor of the formation of a phenotype which is acclimated to higher light intensities. Furthermore, it can be deduced that the RL acclimated state of WT cells is not simply the consequence of missing BL absorption, but represents a discrete acclimation state which requires the presence of AUREO1a. The involvement of BL photoreceptors in the generation of RL phenotypes is not unusual. For example, it was demonstrated that the neutral radical state of an animal-like cryptochrome of *Chlamydomonas reinhardtii* is able to absorb both BL and RL [58]. However, the biochemical properties of the AUREO1a LOV-domain do not allow the generation of a radical state of the chromophore which would be required for RL absorption. Accordingly, the absorption of the AUREO1a of *P. tricornutum* was shown to be restricted to wavelengths in the blue range ([26] and T. Kottke, personal communication).

Therefore, other interaction mechanisms between AUREO1a and RL perception pathways have to be assumed. Recently, protein complexes containing RL absorbing phytochromes and BL absorbing phototropins were discovered in the plasma membranes of *Physcomitrella patens* and *Arabidopsis thaliana* [59]. These protein complexes were shown to be essential for full functionality of phytochromes explaining the loss of RL induced chloroplast movement in phototropin deficient strains of *P. patens* described earlier [60]. Due to the absence of phototropin photoreceptors in diatoms, it can be speculated that AUREO1a may functionally substitute phototropin as interaction partner of phytochrome. This would make AUREO1a essential for a correct function of phytochromes and thus, for RL induced signalling in *P. tricornutum*. The observed additional cytosolic localisation of AUREO1a would in principle allow an interaction with plasma membrane bound phytochromes.

In addition to the physical interaction of phototropins and phytochromes, various other interaction mechanisms between BL and RL perception pathways were described for higher plants and for green algae. For example, it was shown that both phytochromes and cryptochromes regulate the expression of certain components of the phototropin signalling pathway [61], [62], [63] or alter their cellular location [64]. Furthermore, the protein phytochrome kinase substrate 4 was shown to be substrate of both phytochromes and phototropins [65]. If indeed an interaction between AUREO1a and a phytochrome would occur in *P. tricornutum*, the phenotype of *aureo1a* and WT cultures should change upon adding far red radiation to either BL or RL conditions, respectively. An interaction between AUREO1a and a RL perception pathway could be involved in the perception of the BL/RL ratio. This ratio may vary enormously in the euphotic zones of the natural habitats of diatoms [5] and it was shown to correlate comparatively well with the ambient light intensity [6], [66]. Therefore, a putative interaction between AUREO1a and a RL perception pathway might enable the diatoms to combine sensing of light qualities with the ability to integrate the perceived light intensities into a total light intensity perception allowing the cells to acclimate better to their environment. Alternatively, it is possible that the altered phenotypes of the transformed cell lines under both BL and RL conditions result from a light-independent functionality of AUREO1a. However, there is convincing evidence that AUREO1a acts as BL activated transcription factor for the *dsCYC2* gene in *P. tricornutum* [27]. Furthermore, AUREO1a LOVα reacts with conformational changes in response to BL exposure allowing homodimerisation and enhanced DNA binding [26]. Thus it seems likely that the observed light dependent physiological effects originate primarily from a BL induced functionality of AUREO1a.

One important feature of our work is the finding that, although four different aureochromes are expressed in *P. tricornutum*, the silencing of a single aureochrome gene cannot be compensated. This indicates that the individual aureochromes might have discrete functions similar to the AUREO1 and 2 proteins of *V. frigida* [23]. This is further supported by the observation of differential circadian gene expression patterns of the aureochromes of *Thalassiosira pseudonana* [67] and by our finding of four distinct groups of aureochromes thar feature group specific homologous regions. Group 1 and 2 correspond to the respective aureochromes of *V. frigida* described by Takahashi et al. [23], while the other two groups 3 and 4 are dominated by diatom aureochromes, raising the question whether diatoms might possess exclusive classes of aureochromes associated with diatom specific functions. This notion is also supported by the observation that *P. tricornutum*, like the diatoms *P. multiseries* and *F. cylindrus*, possesses at least one aureochrome of each group. If this pattern is confirmed in future research, a re-evaluation of the current aureochrome nomenclature might be appropriate.

A preceding study on the physiological characterisation of *P. tricornutum* in response to different light qualities [28] revealed that the quantum requirement of biomass production was significantly increased in WT cells under ML RL conditions compared to all other tested culture conditions. Although monochromatic RL conditions are artificial and do not occur naturally, this demonstrated that chromatic acclimation can affect the overall cellular energy balance. In contrast to WT cultures, an increased quantum requirement under ML RL conditions compared to other applied culturing conditions was not detected in *aureo1a* cultures. This is in agreement with the apparent acclimation of the aureochrome 1a silenced strains to increased light intensities at ML RL conditions as indicated by elevated NPQ capacity, P_max_ or xanthophyll cycle pigment concentrations. Generally, no obvious disadvantage of *aureo1a* cultures compared to WT cells was detected using our specific experimental setup with persistent exponential growth. However, WT and *aureo1a* cultures were additionally grown in batch cultures revealing that aureochrome 1a silenced strains show a prolonged lag phase during batch cultivation compared to WT cultures, while no significant differences of growth rates during the exponential growth phase were detected (Experiment S1). This indicates that the suppression of AUREO1a in *P. tricornutum* disturbs the initial photoacclimation after changes of the ambient light conditions rather than the growth performance under steady state conditions. Thus, one role of AUREO1a could be the promotion of acclimation to fast changes of ambient light conditions, which are common in the natural habitat of diatoms [5]. In this context it would be interesting to study the performance of *aureo1a* cultures under fluctuating light conditions.

## Supporting Information

Figure S1 **Alignment of 32 aureochromes of twelve different stramenopiles which was used for the generation of a phylogenetic tree by PhyML calculation.**
(PDF)

Figure S2 **Orthoview confocal laser scanning microscopy pictures for visualisation of nuclear co-localisation of aureochrome fusion proteins.**
(PDF)

Figure S3 **Sequence of the synthetic gene used for RNAi-construct generation.**
(PDF)

Figure S4 **Vector map of modified pPha-NR used as scaffold for silencing construct generation.**
(PDF)

Figure S5 **Nuclear localisation sequence (NLS) prediction by the NLStradamus application of the four** ***Phaeodactylum tricornutum*** **aureochromes.**
(PDF)

Figure S6 **Epifluorescence microscopy images of different aureochrome 1a-GFP fusion proteins to visualise dual localisation in nucleus and cytosol.**
(PDF)

Figure S7 **bZIP and LOV domain conservation in** ***P. tricornutum*** **aureochromes.**
(PDF)

Table S1 **Primers used for amplification of aureochromes from** ***P. tricornutum*** **cDNA and verification and sequencing of the aureochrome silencing transformants.**
(PDF)

Table S2 **Comparison of** ***Phaeodactylum tricornutum*** **aureochrome gene models.**
(PDF)

Experiment S1 **Growth curves of WT,** ***aureo1a-15*** **and** ***aureo1a-50*** **batch cultures grown under different light conditions.**
(PDF)

## References

[1] Geider RJ, Delucia EH, Falkowski PG, Finzi AC, Grime JP, et al (2001) Primary productivity of planet earth: biological determinants and physical constraints in terrestrial and aquatic habitats. Global Change Biology 7(8): 849–882.

[2] Nelson DM, Treguer P, Brzezinski MA, Leynaert A, Queguiner B (1995) Production and dissolution of biogenic silica in the ocean - revised global estimates, comparison with regional data and relationship to biogenic sedimentation. Glob Biogeochem Cycle 9(3): 359–372.

[3] Armbrust EV (2009) The life of diatoms in the world’s oceans. Nature 459(7244): 185–192. 19444204 10.1038/nature08057

[4] MacIntyre HL, Kana TM, Geider RJ (2000) The effect of water motion on short-term rates of photosynthesis by marine phytoplankton. Trends in Plant Science 5(1): 12–17. 10637656 10.1016/s1360-1385(99)01504-6

[5] Kirk JTO (2011) Light & photosynthesis in aquatic ecosystems. Cambridge: Cambridge University Press.

[6] Ragni M, D’Alcala MR (2004) Light as an information carrier underwater. Journal of Plankton Research 26(4): 433–443.

[7] Brunet C, Lavaud J (2010) Can the xanthophyll cycle help extract the essence of the microalgal functional response to a variable light environment? Journal of Plankton Research 32(12): 1609–1617.

[8] Lavaud J, Strzepek RF, Kroth PG (2007) Photoprotection capacity differs among diatoms: Possible consequences on the spatial distribution of diatoms related to fluctuations in the underwater light climate. Limnology and Oceanography 52(3): 1188–1194.

[9] Ruban AV, Lavaud J, Rousseau B, Guglielmi G, Horton P, et al (2004) The super-excess energy dissipation in diatom algae: comparative analysis with higher plants. Photosynthesis Research 82(2): 165–175. 16151872 10.1007/s11120-004-1456-1

[10] Depauw FA, Rogato A, d’Alcala MR, Falciatore A (2012) Exploring the molecular basis of responses to light in marine diatoms. Journal of Experimental Botany 63(4): 1575–1591. 22328904 10.1093/jxb/ers005

[11] Margalef R (1978) Life-forms of phytoplankton as survival alternatives in an unstable environment. Oceanol Acta 1(4): 493–509.

[12] Goss R, Jakob T (2010) Regulation and function of xanthophyll cycle-dependent photoprotection in algae. Photosynthesis Research 106(1–2): 103–122. 20224940 10.1007/s11120-010-9536-x

[13] Apt KE, Kroth-Pancic PG, Grossman AR (1996) Stable nuclear transformation of the diatom *Phaeodactylum tricornutum* . Molecular & General Genetics 252(5): 572–579. 8914518 10.1007/BF02172403

[14] Armbrust EV, Berges JA, Bowler C, Green BR, Martinez D, et al (2004) The genome of the diatom *Thalassiosira pseudonana*: Ecology, evolution, and metabolism. Science 306(5693): 79–86. 15459382 10.1126/science.1101156

[15] Bowler C, Allen AE, Badger JH, Grimwood J, Jabbari K, et al (2008) The *Phaeodactylum* genome reveals the evolutionary history of diatom genomes. Nature 456(7219): 239–244. 18923393 10.1038/nature07410

[16] Bräutigam K, Dietzel L, Pfannschmidt T (2010) Hypothesis: A binary redox control mode as universal regulator of photosynthetic light acclimation. Plant signaling & behavior 5(1): 81–85. 20592819 10.4161/psb.5.1.10294 PMC2835968

[17] Durnford DG, Falkowski PG (1997) Chloroplast redox regulation of nuclear gene transcription during photoacclimation. Photosynthesis Research 53(2–3): 229–241.

[18] Foyer CH, Neukermans J, Queval G, Noctor G, Harbinson J (2012) Photosynthetic control of electron transport and the regulation of gene expression. Journal of Experimental Botany 63(4): 1637–1661. 22371324 10.1093/jxb/ers013

[19] Bräutigam K, Dietzel L, Kleine T, Stroeher E, Wormuth D, et al (2009) Dynamic plastid redox signals integrate gene expression and metabolism to induce distinct metabolic states in photosynthetic acclimation in *Arabidopsis* . Plant Cell 21(9): 2715–2732. 19737978 10.1105/tpc.108.062018 PMC2768923

[20] Li Z, Wakao S, Fischer BB, Niyogi KK (2009) Sensing and responding to excess light. Annual Review of Plant Biology. 239–260. 10.1146/annurev.arplant.58.032806.103844 19575582

[21] Walters RG, Rogers JJM, Shephard F, Horton P (1999) Acclimation of *Arabidopsis thaliana* to the light environment: the role of photoreceptors. Planta 209(4): 517–527. 10550634 10.1007/s004250050756

[22] Coesel S, Mangogna M, Ishikawa T, Heijde M, Rogato A, et al (2009) Diatom PtCPF1 is a new cryptochrome/photolyase family member with DNA repair and transcription regulation activity. Embo Reports 10(6): 655–661. 19424294 10.1038/embor.2009.59 PMC2711838

[23] Takahashi F, Yamagata D, Ishikawa M, Fukamatsu Y, Ogura Y, et al (2007) AUREOCHROME, a photoreceptor required for photomorphogenesis in stramenopiles. Proceedings of the National Academy of Sciences of the United States of America 104(49): 19625–19630. 18003911 10.1073/pnas.0707692104 PMC2148339

[24] Suetsugu N, Wada M (2013) Evolution of three LOV blue light receptor families in green plants and photosynthetic stramenopiles: Phototropin, ZTL/FKF1/LKP2 and Aureochrome. Plant & cell physiology 54(1): 8–23. 23220691 10.1093/pcp/pcs165

[25] Ishikawa M, Takahashi F, Nozaki H, Nagasato C, Motomura T, et al (2009) Distribution and phylogeny of the blue light receptors aureochromes in eukaryotes. Planta (Berlin) 230(3): 543–552. 19544070 10.1007/s00425-009-0967-6

[26] Herman E, Sachse M, Kroth PG, Kottke T (2013) Blue-light-induced unfolding of the Jα helix allows for the dimerization of aureochrome-LOV from the diatom *Phaeodactylum tricornutum* . Biochemistry 52(18): 3094–3101. 23621750 10.1021/bi400197u

[27] Huysman MJJ, Fortunato AE, Matthijs M, Schellenberger Costa B, Vanderhaeghen R, et al (2013) AUREOCHROME1a-mediated induction of the diatom-specific Cyclin dsCYC2 controls the onset of cell division in diatoms (*Phaeodactylum tricornutum*). Plant Cell 25(1): 215–228. 23292736 10.1105/tpc.112.106377 PMC3584536

[28] Schellenberger Costa B, Jungandreas A, Jakob T, Weisheit W, Mittag M, et al (2013) Blue light is essential for high light acclimation and photoprotection in the diatom *Phaeodactylum tricornutum* . Journal of experimental botany 64(2): 483–493. 23183259 10.1093/jxb/ers340 PMC3542041

[29] Guindon S, Dufayard J-F, Lefort V, Anisimova M, Hordijk W, et al (2010) New algorithms and methods to estimate Maximum-Likelihood phylogenies: assessing the performance of PhyML 3.0. Systematic Biology 59(3): 307–321. 20525638 10.1093/sysbio/syq010

[30] Le SQ, Gascuel O (2008) An improved general amino acid replacement matrix. Molecular Biology and Evolution 25(7): 1307–1320. 18367465 10.1093/molbev/msn067

[31] Guillard RR, Lorenzen CJ (1972) Yellow-green algae with chlorophyllide *c* . Journal of Phycology 8(1): 10–14.

[32] Mullis K, Faloona F, Scharf S, Saiki R, Horn G, et al (1986) Specific enzymatic amplification of DNA *in vitro* - the polymerase chain-reaction. Cold Spring Harbor Symposia on Quantitative Biology 51: 263–273. 3472723 10.1101/sqb.1986.051.01.032

[33] Zaslavskaia LA, Lippmeier JC, Kroth PG, Grossman AR, Apt KE (2000) Transformation of the diatom *Phaeodactylum tricornutum* (Bacillariophyceae) with a variety of selectable marker and reporter genes. Journal of Phycology 36(2): 379–386.

[34] Gruber A, Vugrinec S, Hempel F, Gould SB, Maier U-G, et al (2007) Protein targeting into complex diatom plastids: functional characterisation of a specific targeting motif. Plant Molecular Biology 64(5): 519–530. 17484021 10.1007/s11103-007-9171-x

[35] Kroth PG (2007) Genetic transformation: a tool to study protein targeting in diatoms. Methods in molecular biology (Clifton, NJ) 390: 257–267. 17951693

[36] Stork S, Moog D, Przyborski JM, Wilhelmi I, Zauner S, et al (2012) Distribution of the SELMA translocon in secondary plastids of red algal origin and predicted uncoupling of ubiquitin-dependent translocation from degradation. Eukaryotic Cell 11(12): 1472–1481. 23042132 10.1128/EC.00183-12 PMC3536273

[37] Poulsen N, Chesley PM, Kröger N (2006) Molecular genetic manipulation of the diatom *Thalassiosira pseudonana* (Bacillariophyceae). Journal of Phycology 42(5): 1059–1065.

[38] Poulsen N, Kröger N (2005) A new molecular tool for transgenic diatoms - Control of mRNA and protein biosynthesis by an inducible promoter-terminator cassette. Febs Journal 272(13): 3413–3423. 15978046 10.1111/j.1742-4658.2005.04760.x

[39] Laemmli UK (1970) Cleavage of structural proteins during assembly of head of bacteriophage-T4. Nature 227(5259): 680–685. 5432063 10.1038/227680a0

[40] Gallagher S, Chakavarti D (2008) Immunoblot analysis. Journal of visualized experiments : JoVE(16). 10.3791/759 PMC2583035 19066547

[41] Gilbert M, Wilhelm C, Richter M (2000) Bio-optical modelling of oxygen evolution using in vivo fluorescence: Comparison of measured and calculated photosynthesis/irradiance (P-I) curves in four representative phytoplankton species. Journal of Plant Physiology 157(3): 307–314.

[42] Wagner H, Jakob T, Wilhelm C (2006) Balancing the energy flow from captured light to biomass under fluctuating light conditions. New Phytologist 169(1): 95–108. 16390422 10.1111/j.1469-8137.2005.01550.x

[43] Jeffrey SW, Humphrey GF (1975) New spectrophotometric equations for determining chlorophylls *a*, *b*, *c1* and *c2* in higher plants, algae and natural phytoplankton. Biochemie und Physiologie der Pflanzen 167(2): 191–194.

[44] Su W, Jakob T, Wilhelm C (2012) The impact of non-photochemical quenching of fluorescence on the photon balance in diatoms under dynamic light conditions. Journal of Phycology 48: 336–346. 27009723 10.1111/j.1529-8817.2012.01128.x

[45] Bilger W, Bjorkman O (1990) Role of the xanthophyll cycle in photoprotection elucidated by measurements of light-induced absorbency changes, fluorescence and photosynthesis in leaves of *Hedera canariensis* . Photosynthesis Research 25(3): 173–185. 24420348 10.1007/BF00033159

[46] Ba ANN, Pogoutse A, Provart N, Moses AM (2009) NLStradamus: a simple Hidden Markov Model for nuclear localization signal prediction. Bmc Bioinformatics 10. 10.1186/1471-2105-10-202 PMC2711084 19563654

[47] Bendtsen JD, Nielsen H, von Heijne G, Brunak S (2004) Improved prediction of signal peptides: SignalP 3.0. Journal of Molecular Biology 340(4): 783–795. 15223320 10.1016/j.jmb.2004.05.028

[48] Lavaud J, Materna AC, Sturm S, Vugrinec S, Kroth PG (2012) Silencing of the violaxanthin de-epoxidase dene in the diatom *Phaeodactylum tricornutum* reduces diatoxanthin synthesis and non-photochemical quenching. Plos One 7(5). 10.1371/journal.pone.0036806 PMC3356336 22629333

[49] Lepetit B, Volke D, Gilbert M, Wilhelm C, Goss R (2010) Evidence for the existence of one antenna-associated, lipid-dissolved and two protein-bound pools of diadinoxanthin cycle pigments in diatoms. Plant Physiology 154(4): 1905–1920. 20935178 10.1104/pp.110.166454 PMC2996015

[50] Schumann A, Goss R, Jakob T, Wilhelm C (2007) Investigation of the quenching efficiency of diatoxanthin in cells of *Phaeodactylum tricornutum* (Bacillariophyceae) with different pool sizes of xanthophyll cycle pigments. Phycologia 46(1): 113–117.

[51] Anning T, MacIntyre HL, Pratt SM, Sammes PJ, Gibb S, et al (2000) Photoacclimation in the marine diatom *Skeletonema costatum* . Limnology and Oceanography 45(8): 1807–1817.

[52] Beer A, Juhas M, Buechel C (2011) Influence of different light intensities and different iron nutrition on the photosynthetic apparatus in the diatom *Cyclotella meneghiniana* (Bacillariophyceae). Journal of Phycology 47(6): 1266–1273. 27020350 10.1111/j.1529-8817.2011.01060.x

[53] Bailleul B, Rogato A, de Martino A, Coesel S, Cardol P, et al (2010) An atypical member of the light-harvesting complex stress-related protein family modulates diatom responses to light. Proceedings of the National Academy of Sciences of the United States of America 107(42): 18214–18219. 20921421 10.1073/pnas.1007703107 PMC2964204

[54] Nymark M, Valle KC, Brembu T, Hancke K, Winge P et al. (2009) An integrated analysis of molecular acclimation to high light in the marine diatom *Phaeodactylum tricornutum*. Plos One 4(11). 10.1371/journal.pone.0007743 PMC2766053 19888450

[55] Sturm S, Engelken J, Gruber A, Vugrinec S, Kroth PG et al.. (2013) A novel type of light-harvesting antenna protein of red algal origin in algae with secondary plastids. BMC Evolutionary Biology 13(159): doi:10.1186/1471-2148-1113-1159. 10.1186/1471-2148-13-159 PMC3750529 23899289

[56] Lepetit B, Sturm S, Rogato A, Gruber A, Sachse M, et al (2013) High light acclimation in the secondary plastids containing diatom *Phaeodactylum tricornutum* is triggered by the redox state of the plastoquinone pool. Plant Physiology 161(2): 853–865. 23209128 10.1104/pp.112.207811 PMC3561024

[57] Nymark M, Valle KC, Hancke K, Winge P, Andresen K et al. (2013) Molecular and photosynthetic responses to prolonged darkness and subsequent acclimation to re-illumination in the diatom *Phaeodactylum tricornutum*. PLoS ONE 8(3). 10.1371/journal.pone.0058722 PMC3592843 23520530

[58] Beel B, Prager K, Spexard M, Sasso S, Weiss D, et al (2012) A flavin binding cryptochrome photoreceptor responds to both blue and red light in *Chlamydomonas reinhardtii* . Plant Cell 24(7): 2992–3008. 22773746 10.1105/tpc.112.098947 PMC3426128

[59] Jaedicke K, Lichtenthaeler AL, Meyberg R, Zeidler M, Hughes J (2012) A phytochrome-phototropin light signaling complex at the plasma membrane. Proceedings of the National Academy of Sciences of the United States of America 109(30): 12231–12236. 22773817 10.1073/pnas.1120203109 PMC3409733

[60] Kasahara M, Kagawa T, Sato Y, Kiyosue T, Wada M (2004) Phototropins mediate blue and red light-induced chloroplast movements in *Physcomitrella patens* . Plant Physiology 135(3): 1388–1397. 15247376 10.1104/pp.104.042705 PMC519056

[61] Lariguet P, Schepens I, Hodgson D, Pedmale UV, Trevisan M, et al (2006) PHYTOCHROME KINASE SUBSTRATE 1 is a phototropin 1 binding protein required for phototropism. Proceedings of the National Academy of Sciences of the United States of America 103(26): 10134–10139. 16777956 10.1073/pnas.0603799103 PMC1502518

[62] Tsuchida-Mayama T, Sakai T, Hanada A, Uehara Y, Asami T, et al (2010) Role of the phytochrome and cryptochrome signaling pathways in hypocotyl phototropism. Plant J 62(4): 653–662. 20202166 10.1111/j.1365-313X.2010.04180.x

[63] Kami C, Hersch M, Trevisan M, Genoud T, Hiltbrunner A, et al (2012) Nuclear phytochrome A signaling promotes phototropism in *Arabidopsis* . Plant Cell 24(2): 566–576. 22374392 10.1105/tpc.111.095083 PMC3315233

[64] Han I-S, Tseng T-S, Eisinger W, Briggs WR (2008) Phytochrome A regulates the intracellular distribution of phototropin 1-green fluorescent protein in *Arabidopsis thaliana* . Plant Cell 20(10): 2835–2847. 18952772 10.1105/tpc.108.059915 PMC2590736

[65] Demarsy E, Schepens I, Okajima K, Hersch M, Bergmann S, et al (2012) Phytochrome Kinase Substrate 4 is phosphorylated by the phototropin 1 photoreceptor. Embo Journal 31(16): 3457–3467. 22781128 10.1038/emboj.2012.186 PMC3419926

[66] Lopez-Figueroa F, Niell FX (1989) Red-light and blue-light photoreceptors controlling chlorophyll *a* synthesis in the red alga *Porphyra umbilicalis* and in the green alga *Ulva rigida* . Physiologia Plantarum 76(3): 391–397.

[67] Ashworth J, Coesel S, Lee A, Armbrust EV, Orellana MV, et al (2013) Genome-wide diel growth state transitions in the diatom *Thalassiosira pseudonana* . Proceedings of the National Academy of Sciences 110(18): 7518–7523. 10.1073/pnas.1300962110 PMC3645528 23596211

