# Correction: Aureochrome 1a Is Involved in the Photoacclimation of the Diatom *Phaeodactylum tricornutum*

**DOI:** 10.1371/annotation/7000208e-7505-4c2d-beed-fc99236bbe9f

**Published:** 2013-09-26

**Authors:** Benjamin Schellenberger Costa, Matthias Sachse, Anne Jungandreas, Carolina Rio Bartulos, Ansgar Gruber, Torsten Jakob, Peter G. Kroth, Christian Wilhelm

There was an error in the Author Contributions. The correct version of this section is available below.

Conceived and designed the experiments: BSC MS AJ AG TJ PGK CW. Performed the experiments: BSC MS AJ CRB AG. Analyzed the data: BSC MS AJ AG TJ PGK CW. Wrote the paper: BSC CRB TJ MS PGK CW. 

