# Supplementary figures and images for: Aureochrome 1a Is Involved in the Photoacclimation of the Diatom Phaeodactylum tricornutum
Source: PLoS One. 2013 Sep 20;8(9):e74451. doi: 10.1371/journal.pone.0074451 (PMC3779222; doi:10.1371/journal.pone.0074451)

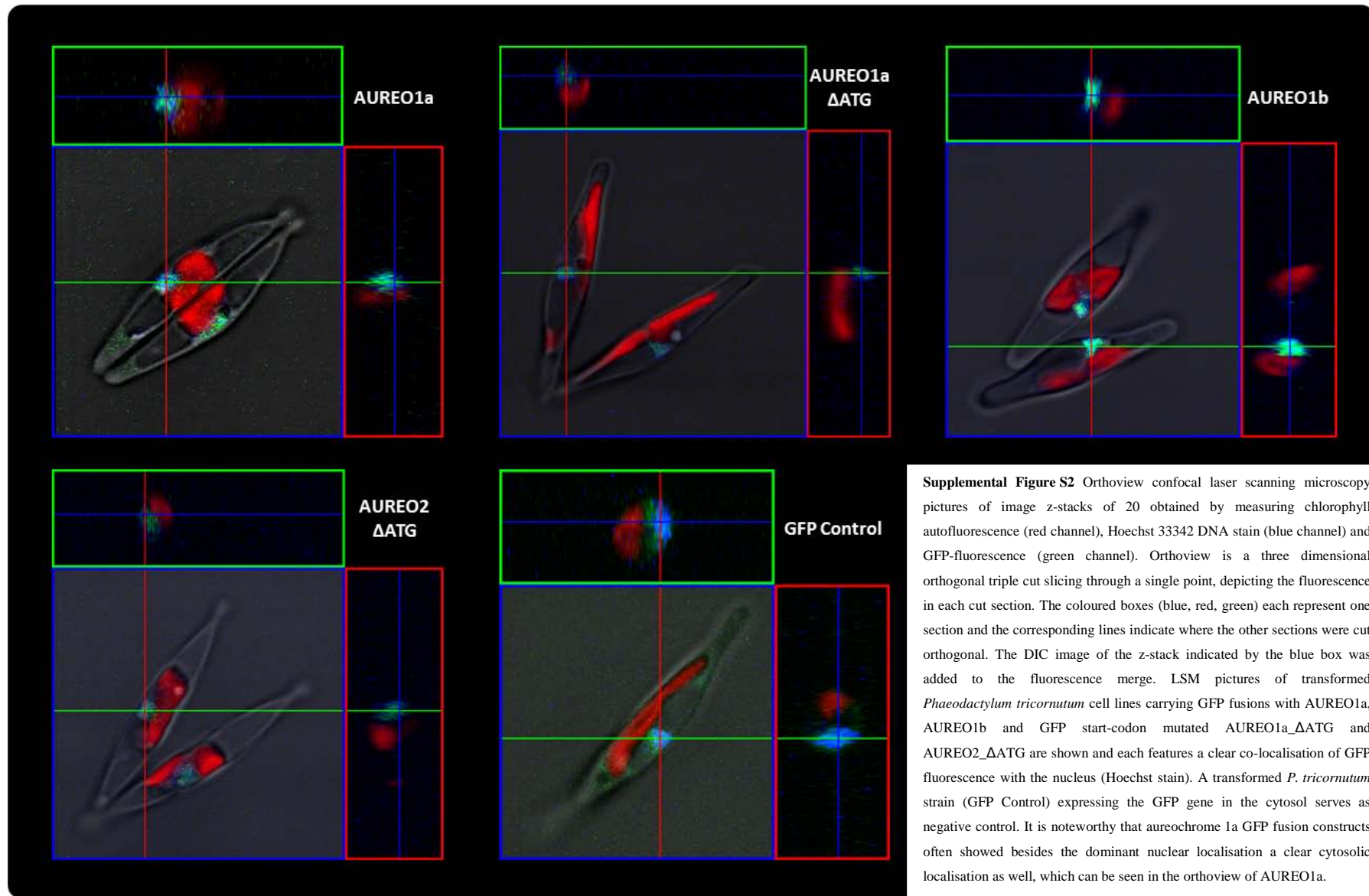

Supplement: Figure S2 — Orthoview confocal laser scanning microscopy pictures for visualisation of nuclear co-localisation of aureochrome fusion proteins. (PDF) [file pone.0074451.s002.pdf]
