# Supplementary material for: Aureochrome 1a Is Involved in the Photoacclimation of the Diatom Phaeodactylum tricornutum
Source: PLoS One. 2013 Sep 20;8(9):e74451. doi: 10.1371/journal.pone.0074451 (PMC3779222; doi:10.1371/journal.pone.0074451)
Supplement: Figure S3 — Sequence of the synthetic gene used for RNAi-construct generation. (PDF) [file pone.0074451.s003.pdf]

5‘-tctagaggtaccTGGAATCAGTTCTTCATTGCTGCCTTGCGGGATGCCGGTGGCAACGT
GACCAACTTTGTGGGGGTGCAGTGCAAGGTGTCCGACCAATACGCCGCCACAGTCACCAAGC
AACAGGAAGAAgttaac-3‘

Xba1	(sticky)	t/ctaga
Kpn1	(sticky)	ggtac/c
HpaI	(blunt)	gtt/aac

Supplemental Figure S3 Sequence of synthetic gene for RNAi-construct generation. 120bp fragment of AUREO1a (49116; 982-1101) flanked by Xba1 and Kpn1 sticky restriction sites at the 5’ terminus and HpaI blunt end restriction site at the 3’ terminus.
