# Supplementary material for: Aureochrome 1a Is Involved in the Photoacclimation of the Diatom Phaeodactylum tricornutum
Source: PLoS One. 2013 Sep 20;8(9):e74451. doi: 10.1371/journal.pone.0074451 (PMC3779222; doi:10.1371/journal.pone.0074451)
Supplement: Figure S4 — Vector map of modified pPha-NR used as scaffold for silencing construct generation. (PDF) [file pone.0074451.s004.pdf]

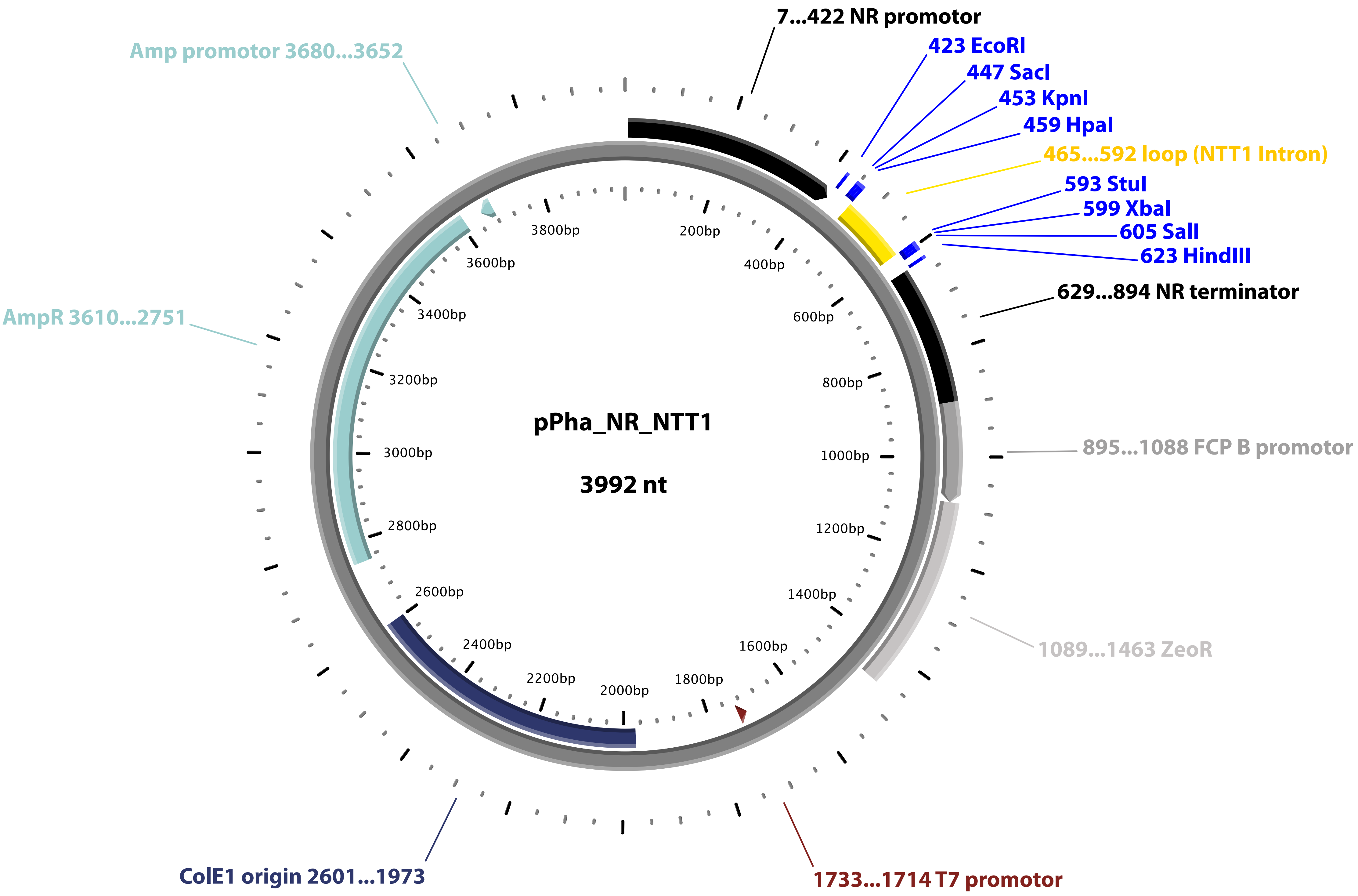


```
7 CGGAAGTGACTGTAAACGAGAAGTGCACGAAGCCTTTTCTTGTGACGTCACAAACCGAACAGCCCTACGTGGCGTGCGACT
TTCCGGCACTTTGGATATCGTTGCCCTATATGTATTGGGTGTATACATGTCGTATTCCAACTACGACAGAAAACAGTCAGTTA
CGGGTATTCGAACTTCCGGCACCTGCAGCGAGGAGTTTTGTGTCCGTCGCACATCCTCCGTGCTTTCGGCACCTACGTAGGCG
GCAAACTTCCTGCCTTCCGCCCGCCTTGCGGCATTCCGGCTCCGGGCCAGAATTGCCCGGGTGTTCACAATTTTGCCTCCTCA
CGAAAAAACGTTCTACTTTGTATTTTGGTCGGGTTTCGGATCCTTCCAGCAACCATTCTCATTCAAAGTCACCACTTGTGCGA
ACGgaattcGATATCATCGACTAATTCgagctcggtaccgttaacGTACGTATCTGCACACATACCAATTCTCCACACCGACA
TGATGCAATTATCATGTTGGTTACTGCGCGCCATACATCGCTCACATACGCTTTCCCTTTTCCTCTCGCGCGATTGCACACTT
TATACAGaggccttctagagtcgacCTGCAGGCATGCaagctt 628
```

EcoRI	(sticky)	g/aattc
SacI	(sticky)	gagct/c
Kpn1	(sticky)	ggtac/c
HpaI	(blunt)	gtt/aac
StuI	(blunt)	agg/cct
Xba1	(sticky)	t/ctaga
SalI	(sticky)	g/tcgac
HindIII	(sticky)	a/agctt

Supplemental Figure S4 : Vector map of modified pPha-NR (GenBank accession no. JN180663). Blunt end StuI and HpaI restriction sites were introduced as well as the second intron of the *P. tricornutum* NTT1 gene (49533), which serves as loop domain of the silencing mRNA. The sequence of the region of interest is given. The NR promoter sequence is marked in black and the NTT1 intron sequence in yellow. All usable restriction sites for RNAi construct generation are marked as colour coded small letter sequences. For cloning purposes a mixed digest with blunt and sticky end enzymes is performed. When a single sequence with multiple restriction sides on either flank is used for generation of both sense and antisense orientated sequences, it is important to introduce the first sequence with an “inner” and the second by using an “outer” sticky restriction site to prevent introduction of additional restriction sites for enzymes, which shall be utilised for the introduction of the second fragment.
