# Supplementary material for: Aureochrome 1a Is Involved in the Photoacclimation of the Diatom Phaeodactylum tricornutum
Source: PLoS One. 2013 Sep 20;8(9):e74451. doi: 10.1371/journal.pone.0074451 (PMC3779222; doi:10.1371/journal.pone.0074451)
Supplement: Figure S5 — Nuclear localisation sequence (NLS) prediction by the NLStradamus application of the four Phaeodactylum tricornutum aureochromes. (PDF) [file pone.0074451.s005.pdf]

AUREO1a

Predictions for Request Sequence

Viterbi Path

128 - PKKKSSSSSTSGSGSRSDRKMSEQQKVERRERNREHAKRSRIRKK - 172

Viterbi Path

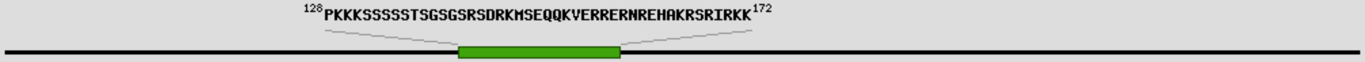

Posterior @ 0.6

129 - KKKSSSSSTSGSGSRSDRKMSEQQKVERRERNREHAKRSRIRKK - 172

Posterior @ 0.6

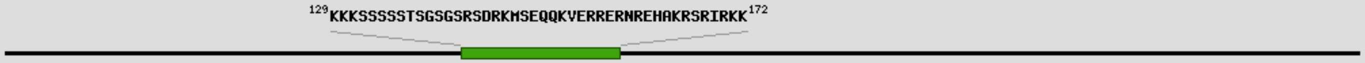

Posterior Graphic. Threshold Marked @ 0.6

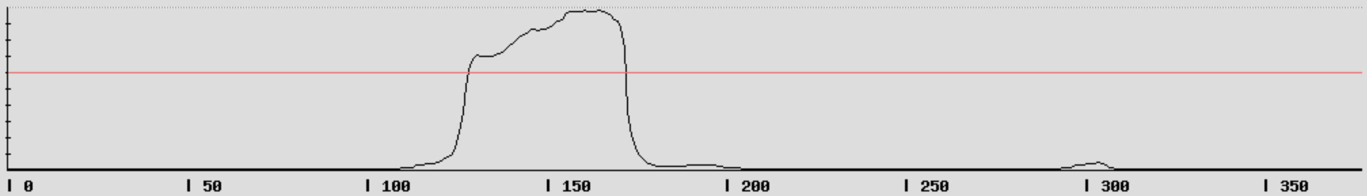

AUREO1b	Predictions for Request Sequence
Viterbi Path	
Viterbi Path	
Posterior @ 0.6	206 - RRQRNREHAKRSRVRKK - 222
Posterior @ 0.6	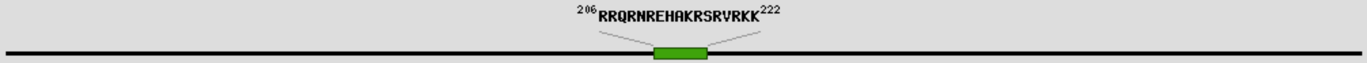
Posterior Graphic. Threshold Marked @ 0.6	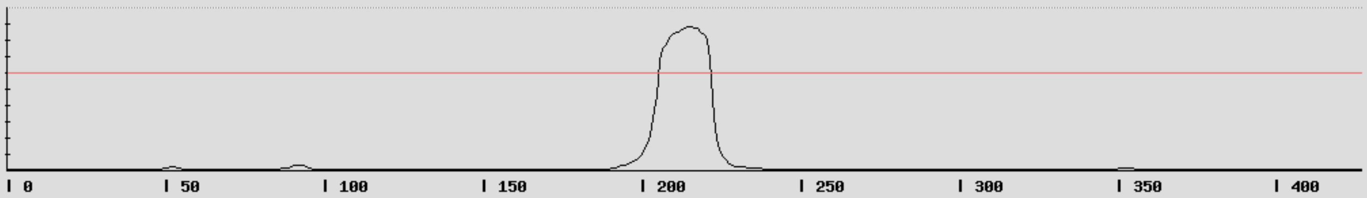

AUREO1c

	Predictions for Request Sequence
Viterbi Path	
Viterbi Path	
Posterior @ 0.6	100 - ERRERNREHAKRSRLRKK - 117
Posterior @ 0.6	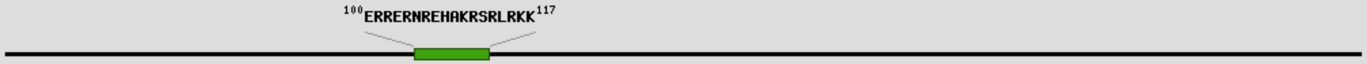
Posterior Graphic. Threshold Marked @ 0.6	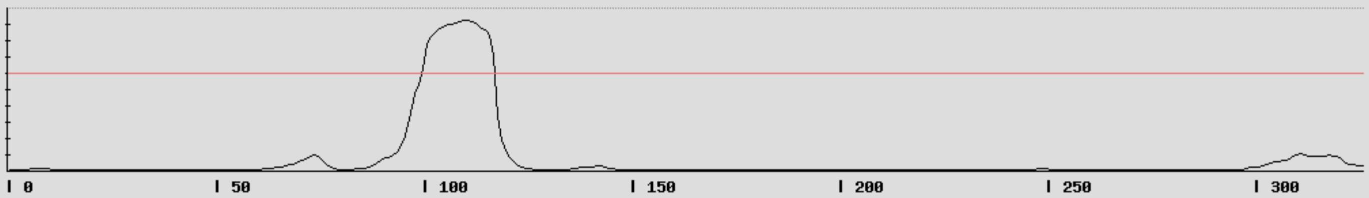

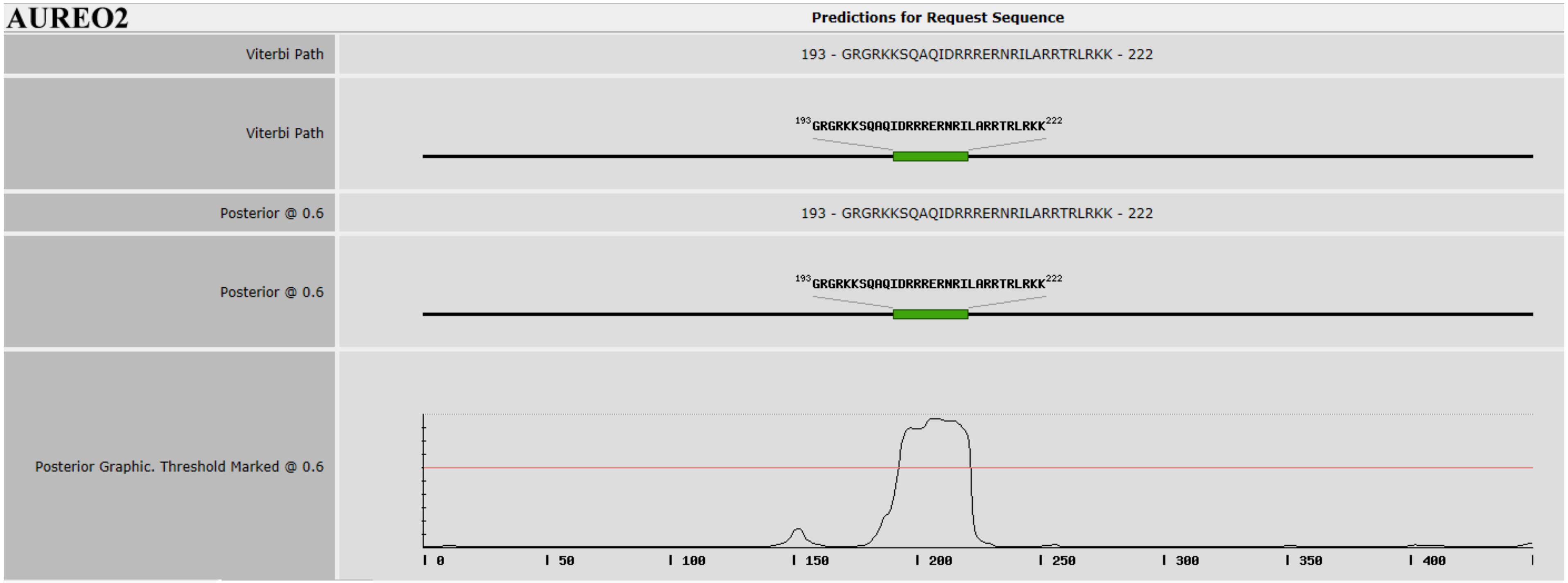

Supplemental Figure S5 Nuclear localisation sequence (NLS) prediction by the NLStradamus application [Φ] of the four *Phaeodactylum tricornutum* aureochromes AUREO1a (49116), AUREO1b (49458), AUREO1c (56742) and AUREO2 (56688). Depicted are the results for 2 state HMM dynamic analyses by Viterbi and Posterior prediction types. For Posterior prediction the default 60 % cut-off was applied. For each of the four aureochromes Posterior type predicts a high probability NLS sequence.

[Φ] http://www.moseslab.csb.utoronto.ca/NLStradamus/; Nguyen Ba AN, Pogoutse A, Provart N, Moses AM. **NLStradamus: a simple Hidden Markov Model for nuclear localization signal prediction.** *BMC Bioinformatics.* 2009 Jun 29;**10(1)**:202.
