# Supplementary material for: Aureochrome 1a Is Involved in the Photoacclimation of the Diatom Phaeodactylum tricornutum
Source: PLoS One. 2013 Sep 20;8(9):e74451. doi: 10.1371/journal.pone.0074451 (PMC3779222; doi:10.1371/journal.pone.0074451)
Supplement: Figure S6 — Epifluorescence microscopy images of different aureochrome 1a-GFP fusion proteins to visualise dual localisation in nucleus and cytosol. (PDF) [file pone.0074451.s006.pdf]

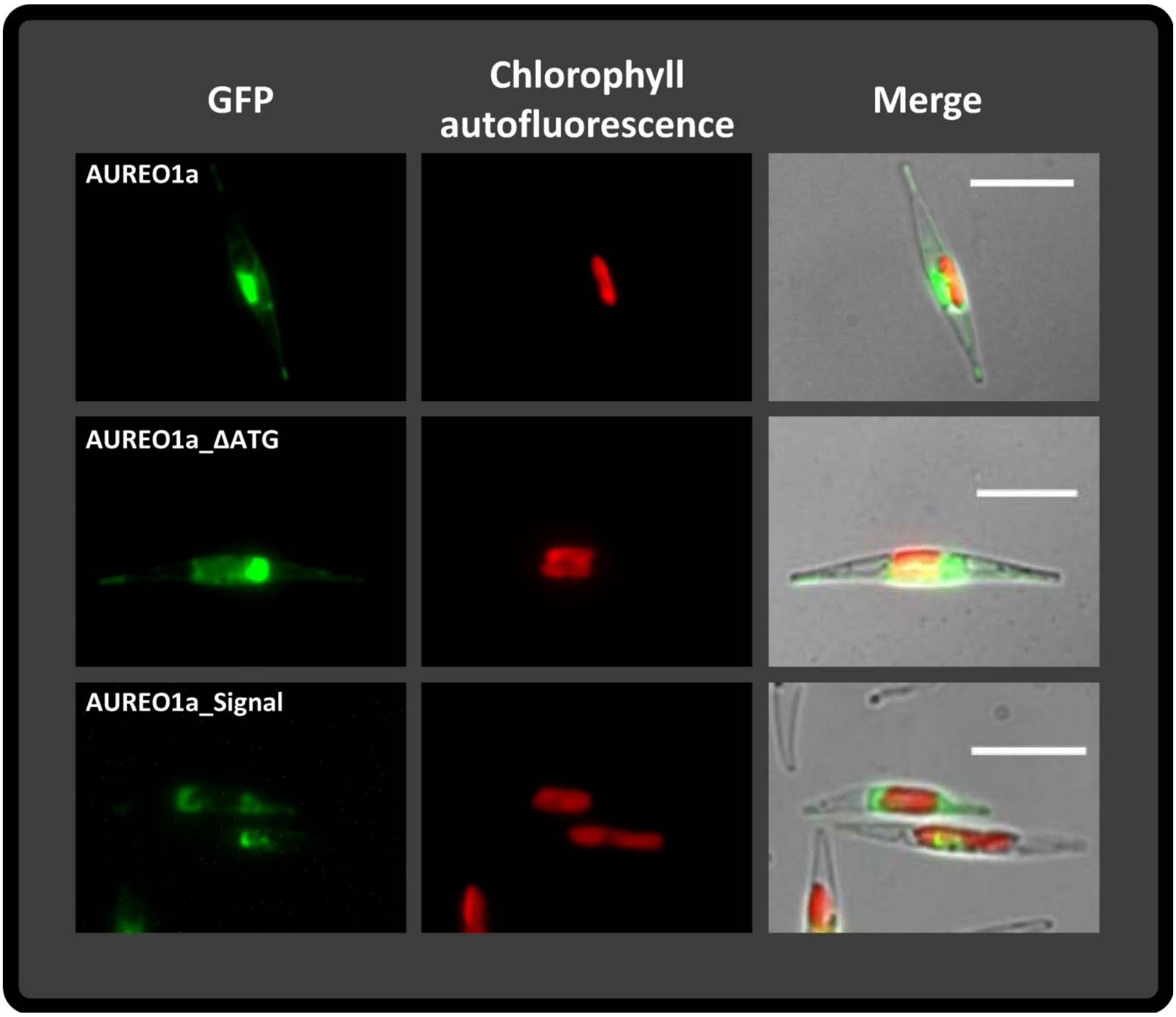

Supplemental figure S6: Epifluorescence microscopy images of different aureochrome 1a-GFP fusion proteins (AUREO1a (49116), AUREO1a_ΔATG (49116) and AUREO1a_Signal (56684)) are shown. From left to right: GFP fluorescence (green), chlorophyll autofluorescence (red) and a merge of both channels with the corresponding DIC image. The white scale bars correspond to 10 µm. All aureochrome 1a fusion proteins feature distinct nuclear localisation and additional cytosolic signals.
