# Supplementary material for: Aureochrome 1a Is Involved in the Photoacclimation of the Diatom Phaeodactylum tricornutum
Source: PLoS One. 2013 Sep 20;8(9):e74451. doi: 10.1371/journal.pone.0074451 (PMC3779222; doi:10.1371/journal.pone.0074451)
Supplement: Figure S7 — bZIP and LOV domain conservation in P. tricornutum aureochromes. (PDF) [file pone.0074451.s007.pdf]

```
CLUSTAL 2.1 multiple sequence alignment

AUREO1a     -----------------------------MTDNNKSLSAHAQAA----VT 17
AUREO1b     -MDDFDLNEIFAEYFTNEFDDPLNAYTSSMANHNGAVTAVAMTQNDTVPT 49
AUREO1c     ------------------------------------MADQAKAN----PS 10
AUREO2      MAQNLQMPRFGRANNTSGADTWGDCDAFDVDMLTEYLLNDGTLTSSGVTF 50
                                                :   .

AUREO1a     -----NRGNPATLNLDDIFGDVMFTPDGDTVFMSEQKEELLNSGEREVTT 62
AUREO1b     EGRKLAQTGGLTLPTGGIRTTFHTAAIQKAPLITDGTNPVTKKQKTDEQS 99
AUREO1c     -----GPSNTAPL-----------------FQMSD--------------- 23
AUREO2      DFNMDGAAHLSSTVSPENSEDGALPTVADSNEISAEVQKFAASHEGPSYV 100
                       .                    ::

AUREO1a     MASKATQDGQYQPVQQGGGLYTTQLYDNS---KPALTMGVAGGINVQAT- 108
AUREO1b     QQQHQQLLHVQNPLQQQQQQALAAAHNS----AMQAHQQTQNASNMQHPG 145
AUREO1c     ------------PANPSSTLDLDDLFND------IYYMDLPNGSNMDS-- 53
AUREO2      AVATSGMPIAPSPSLSFPALPTQVSMPGNDGQGLVFHHHQQQHANSTATS 150
                        *              .                *

AUREO1a     --------APVPYKSAPQATHHLQYAAPKKKSSSSSTSGSGS----RSDR 146
AUREO1b     AAMVQGGMVSLPVGVGIRLGGIGGIAPATAQSATRTSGVPGQFNMWGPGT 195
AUREO1c     --------DPTPVD-VPSSNGNGHDGASKKRSADDFDGDSLD----GTGN 90
AUREO2      KRR-----RIDGMSGSALLAVSGGPTGSAFLGGDQLAAAAARAIQNQGRG 195
                                       .   ..    . .

AUREO1a     K-MSEQQKVERRERNREHAKRSRIRKKFLLESLQQSVSLLKEENEKLKTS 195
AUREO1b     GGMSEQAVAERRQRNREHAKRSRVRKKFMLESLQEQVREMQKQNQNLRML 245
AUREO1c     KDLTEQQKLERRERNREHAKRSRLRKKFLLESLQEQIHGLEEQLDGLKSA 140
AUREO2      RKKSQAQIDRRRERNRILARRTRLRKKFFFESLQKEIMDLQRENLVLKEL 245
               ::    .**:***  *:*:*:****::****:.:  ::.:   *:

AUREO1a     IRSHLGDEKADTLIDSANNNKTDVDGLLASSQGIANKVLDDPDFSFIKAL 245
AUREO1b     VQEHIP-EHAMKIIAECCTSSPLFEEMDGIDQTKGAN-LERADFSLMQSL 293
AUREO1c     IKKELP-QQAEQIITRICGDKEKFTPLPMPSGFGPVKTLMEPDFRLMSAL 189
AUREO2      VKVNISGEEGKKILEGCNAAENLPSSVLEACG--EENDMDSQDFNLVRSI 293
            :: .:  :..  ::      .     :         : :   ** :: ::

AUREO1a     QTAQQNFVVTDPSLPDNPIVYASQGFLNLTGYSLDQILGRNCRFLQGPET 295
AUREO1b     TMGQQCFVLSDPKLPDNPIVFASPGFYKLTGYTSREVLGRNCRFLQGPGT 343
AUREO1c     SGSQQNFAISDPTLPDNPIVYVSQGFLDLTGYTLDQVLGRNCRFLQGPGT 239
AUREO2      QSSQHSFMITDPSLQDNPIVFASDDFLKLTGYTREQVLGRNCRFLQGTET 343
              .*: * ::**.* *****:.* .* .****:  ::**********. *

AUREO1a     DPKAVERIRKAIEQGNDMSVCLLNYRVDGTTFWNQFFIAALRDAGGNVTN 345
AUREO1b     DAKAVDVIRKAVGTGSDATVCLLNYKADGTPFWNQFFIAALRDSDNCIVN 393
AUREO1c     DQSAVEVIRKGITEGVDTSVCLLNYKADGTPFWNQFFVASLRDAENNVVN 289
AUREO2      SQEKVNQIRKNLSEGEDVTVTLMNYTADGTPFWNKLFIAALRDAQNNIVN 393
            . . *: *** :  * * :* *:** .***.***::*:*:***: . :.*

AUREO1a     FVGVQCKVSDQYAATVTKQQEEEEEAAANDDED----------------- 378
AUREO1b     YVGVQTEVEP--QAGVSMLEDKVNAILPLQTKDDSSE------------- 428
AUREO1c     HVGVQCEVS---KAVVEKHMGEQKKAAAEAAKARPVTTTSS--------- 327
AUREO2      FIGVIVKVARPEPGDPEHDKGNESEQNDGDESDEDAEDTVRAIEGAVTAA 443
            .:**  :*     .       : .       .

AUREO1a     --------
AUREO1b     --------
AUREO1c     --------
AUREO2      VAAAGRSS 451
```

Supplemental figure S7: Protein sequence alignment of the four *P. tricornutum* aureochromes The bZIP and LOV domains are colour coded in analogy to the domain definition of Takahashi et al (2007) Φ based on *Vaucheria frigida*, *Thalassiosira pseudonana* and *Fuchus distichus* aureochromes: the corresponding highly conserved amino acids of the

bZIP domain are layered red and of the LOV domain bright blue. Conserved amino acids for both domains are marked in yellow. Besides high homology in the bZIP and LOV domain *P. tricornutum* aureochromes exhibit no strong overall homologies which might indicate different effector roles for each aureochrome. AUREO1b features four deviations in the highly conserved amino acids of the bZIP DNA binding domain (marked with white letters on blue ground). This raises the question if AUREO1b is generally able to bind DNA and if yes, it is likely to bind a distinctly different DNA motif compared to other aureochromes.

[Φ] Takahashi F Yamagata D, Ishikawa M, Fukamatsu Y, Ogura Y et al. (2007) AUREOCHROME, a photoreceptor required for photomorphogenesis in stramenopiles. Proceedings of the National Academy of Sciences of the United States of America 104(49): 19625-19630.
