# Supplementary material for: Aureochrome 1a Is Involved in the Photoacclimation of the Diatom Phaeodactylum tricornutum
Source: PLoS One. 2013 Sep 20;8(9):e74451. doi: 10.1371/journal.pone.0074451 (PMC3779222; doi:10.1371/journal.pone.0074451)
Supplement: Table S1 — Primers used for amplification of aureochromes from P. tricornutum cDNA and verification and sequencing of the aureochrome silencing transformants. (PDF) [file pone.0074451.s008.pdf]

Target	Abbrev.	Protein ID (JGI[1])		Primersequence 5'→ 3'	Amplicon Length [bp]	T_m [2]	Comments
Aureochrome 1a short	*AUREO1a*	49116	for	CATTTCATTACCATGACCGAC	1146	57.7 °C	full length without STOP (→ GFP fusion)
			rev	GTCTTCGTCATCGTTGGC		57.8 °C	
Aureochrome 1a long (with putative signalpeptide)	*AUREO1aSig*	56684	for	ATGCCGTCCGGGTTCC	1305	59.3 °C	full length without STOP (→ GFP fusion)
			rev	GTCTTCGTCATCGTTGGC		57.8 °C	
Aureochrome 1b	*AUREO1b*	49458	for	GCTAGATGGATGATTTTGATTTG	1289	61.1 °C	full length without STOP (→ GFP fusion)
			rev	CTCGCTACTATCATCTTTTGTC		57.5 °C	
Aureochrome 2	*AUREO2*	56688	for	GACTTCAATTTCACCATGGCC	1368	59.8 °C	full length without STOP (→ GFP fusion)
			rev	CGACGATCTACCTGCGG		58.0 °C	
RNAi Check	-	-	for	GGTGTTCACAATTTTGCCTCC	109	61.0 °C	for primer binds at NR promotor
			rev	TCTAGAAGGCCTCTGTATAAAGTGTGCAATCGC		73.0 °C	rev primer binds at NTT1; 5' unpaired overhang

1 Joint Genome Institute (http://genome.jgi.doe.gov/Phatr2/Phatr2.home.html)

2 Thermodynamic melting temperatures according to Thermo Scientific REviewer™ (http://www.thermoscientificbio.com/webtools/reviewer/)

Table S1 Primers used for amplification of aureochromes from *P. tricornutum* cDNA and verification and sequencing of the aureochrome silencing transformants
