# Supplementary material for: Aureochrome 1a Is Involved in the Photoacclimation of the Diatom Phaeodactylum tricornutum
Source: PLoS One. 2013 Sep 20;8(9):e74451. doi: 10.1371/journal.pone.0074451 (PMC3779222; doi:10.1371/journal.pone.0074451)
Supplement: Table S2 — Comparison of Phaeodactylum tricornutum aureochrome gene models. (PDF) [file pone.0074451.s009.pdf]

Gene	Protein ID[1]	Alternative gene models[1]	Genomic coordinates	Comment	Intron boundaries	EST support	N-terminal targeting signals
**AUREO1a**	49116		chr_20:631346-632840	N-terminal extension of same reading frame is possible, compare to 56775, 56684, 56683 and 56680	uncertain, see alternative model 56681	full	no
		56775	chr_20:631346-632877	N-terminal extension of 49116	uncertain, see alternative model 56681	partial (intron supported, N-terminus not supported)	no
		56684	chr_20:631335-632907	N-terminal extension of 49116	uncertain, see alternative model 56681	partial (intron supported, N-terminus not supported)	SignalP 3.0[2] predicts signal peptide
		56683	chr_20:631335-632967	N-terminal extension of 49116	uncertain, see alternative model 56681	partial (intron supported, N-terminus not supported)	SignalP 3.0[2] predicts signal peptide in the region of the 56684 N-terminus
		56680	chr_20:631335-633099	N-terminal extension of 49116	uncertain, see alternative model 56681	partial (intron supported, N-terminus not supported)	no
		56681	chr_20:631999-633099	lack of intron results in short reading frame	uncertain, see all other AUREO1a gene models	partial (absence of intron supported in part of the ESTs, N-terminus not supported)	no
**AUREO1b**	49458		chr_22:298435-299975		5' end might have additional intron, compare to 56685	unambiguous except first intron	no
		56685	chr_22:297993-299987		intron at 5' end might be skipped, compare to 49458	unambiguous except first intron	no
**AUREO1c**	56742		chr_8:267171-268406		unambiguous	full	no
**AUREO2**	56688		chr_19:198962-203087	Intron boundaries of first intron differ from alternative gene model, deviations all in 5' UTR, supported by majority of ESTs	uncertain	unambiguous except first intron	no
		56060	chr_19:198962-202710		uncertain, compare to 56688	unambiguous except first intron	no

[1] Protein IDs refer to the DOE Joint Genome Institute *Phaeodactylum tricornutum* genome database v.2.0, see http://genome.jgi-psf.org/Phatr2/Phatr2.home.html, Bowler et al. doi: 10.1038/nature07410.

[2] see http://www.cbs.dtu.dk/services/TargetP/, Bendtsen et al. doi: 10.1016/j.jmb.2004.05.028.

Supplemental Table S2 Comparison of *Phaeodactylum tricornutum* aureochrome gene models
