# Supplementary material for: Aureochrome 1a Is Involved in the Photoacclimation of the Diatom Phaeodactylum tricornutum
Source: PLoS One. 2013 Sep 20;8(9):e74451. doi: 10.1371/journal.pone.0074451 (PMC3779222; doi:10.1371/journal.pone.0074451)
Supplement: Experiment S1 — Growth curves of WT, aureo1a-15 and aureo1a-50 batch cultures grown under different light conditions. (PDF) [file pone.0074451.s010.pdf]

SUPPLEMENTAL EXPERIMENT S1

Materials and methods

For the measurement of growth curves, WT, *aureo1a-15* and *aureo1a-50* cultures were cultivated in air-lifted 100 ml test tubes in f/2 medium according to Guillard and Lorenzen (1972) without silica and with half of the original salt content. Cultures were inoculated with exponentially growing stock cultures adapted to white light with an intensity of 35 µmol photons m^{-2} s^{-1}. Starting cell density was adjusted to 50000 cells ml^{-1} for each culture. LL cultures were illuminated with either 42 µmol photons m^{-2} s^{-1} red light or 24 µmol photons m^{-2} s^{-1} blue light, ML cultures were illuminated with either 124 µmol photons m^{-2} s^{-1} red light or 72 µmol photons m^{-2} s^{-1} blue light for all three strains. In batch cultures, a reliable determination of Q_{Phar} is not possible due to the constant change of the Chl *a* content of the cultures in combination with continuous variations of $a*_{Phy}$. Therefore, identical light intensities were applied for both WT cultures and aureochrome 1a silenced cell lines which stands in contrast to the specifically adjusted cultivation conditions used for semi-continuous cultures (see Table 1). Absorption at 674 nm was recorded daily at the same time using a scattering corrected spectrophotometer (M500, Zeiss, Jena, Germany) adjusted to a bandwidth of 1 nm. Measurements were performed until cultures reached the stationary phase.

Results

Growth curves of WT, *aureo1a-15* and *aureo1a-50* cultures in dependence of the specific illumination conditions are depicted in supplemental figure S1. LL cultures reached the stationary phase after 10 to 11 days. WT cultures possessed a shorter initial lag phase prior to the exponential growth phase than both aureochrome 1a silenced strains. This effect was more pronounced under RL conditions (S1B) than under BL conditions (S1A). At ML conditions, the stationary phase was reached after 5 to 7 days. Again, WT cultures possessed a faster growth compared to *aureo1a* cultures. This effect was more pronounced under RL conditions (S1D) than under BL conditions (S1C).

Discussion

Both aureochrome 1a silenced cell lines exhibited a prolonged lag phase independent of the investigated light qualities. This might indicate that aureochrome 1a is important for the acclimation to new light conditions. For the interpretation of the data, it has to be considered that $a*_{Phy}$ was increased in the aureochrome 1a silenced cell lines cultivated under semi-

continuous conditions under ML (Table 1). Although this parameter was not measured for the batch cultures, it seems possible that ML cultures of aureochrome 1a silenced cells absorbed more photons than the corresponding WT cultures. This would result in an increased energy supply for these cultures. However, an increased energy supply does not support a prolonged lag phase, it should promote a shortened lag phase.

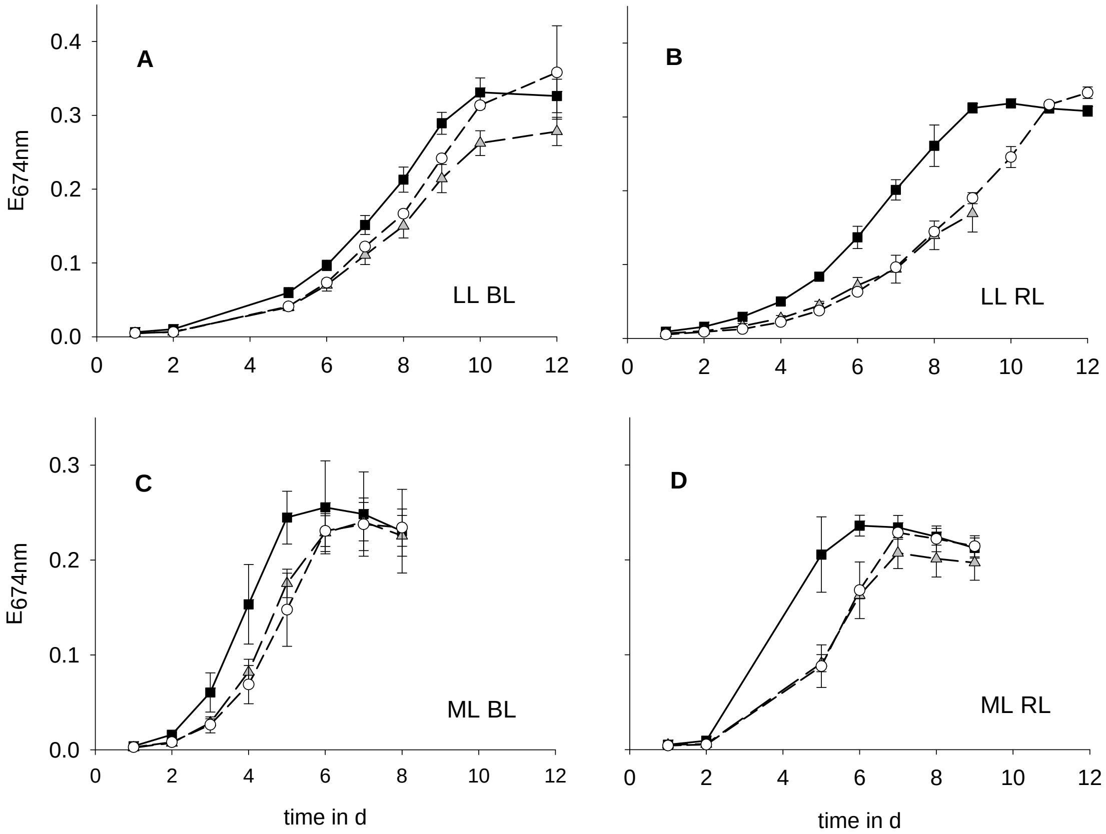

Supplemental Figure S1: Growth curves of WT (black squares), *aureo1a-15* (grey triangles) and *aureo1a-50* (white circles) batch cultures grown under different light conditions. Algae were cultivated under low light (LL) conditions at an incident irradiance of 24 µmol blue photons m^{-2} s^{-1} (A) or 42 µmol red photons m^{-2} s^{-1} (B). Under medium light (ML) conditions, algae were cultivated conditions at an incident irradiance of 72 µmol blue photons m^{-2} s^{-1} (C) or 124 µmol red photons m^{-2} s^{-1} (D). Mean values are shown with standard deviation (n = 3).
