# Supplementary material for: Aureochrome 1a Is Involved in the Photoacclimation of the Diatom Phaeodactylum tricornutum
Source: PLoS One. 2013 Sep 20;8(9):e74451. doi: 10.1371/journal.pone.0074451 (PMC3779222; doi:10.1371/journal.pone.0074451)
Supplement: Figure S1 — Alignment of 32 aureochromes of twelve different stramenopiles which was used for the generation of a phylogenetic tree by PhyML calculation. (PDF) [file pone.0074451.s001.pdf]

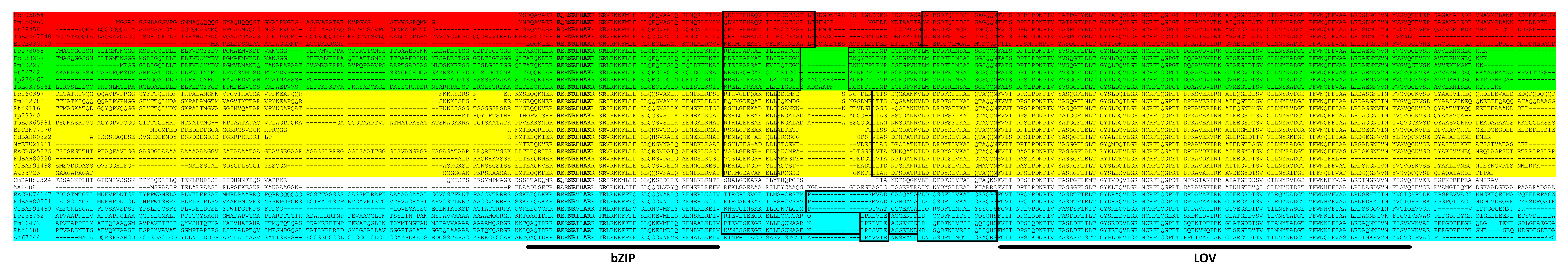

Supplemental Figure S1 Alignment of 32 aureochromes of twelve different stramenopiles used for the generation of a phylogenetic tree by PhyML calculation(http://www.atgc-montpellier.fr/phyml/). The Alignment was manually refined to include mainly comparable homologous regions, resulting in an alignment of 360 amino acid positions in total. bZIP- and LOV domains are marked below the alignment. The six most important amino acid positions for DNA binding according to the NCBI conserved domains database for feature 1 of the bZIP domain (accession number : cd12193: B_zip1) are marked in bold letters. It clearly shows a high conservation of these positions for all investigated aureochromes. Aureochromes clustering into the same group of the phylogenetic tree are highlighted by group specific colours, which correspond to the colouring in the phylogenetic tree (Fig. 2): group 1 yellow, group 2 cyan, group 3 red and group 4 green. The sequences between bZIP and LOV domain exhibit increased group specific homologies which are marked by black boxes. Interestingly in group 2 (cyan) diatoms and other algae share the same homology in proximity to the LOV domain but they show more species specific homologies in the other parts of the sequence. Here, the ditatom aureochromes are highly homologous in the whole region between bZIP and LOV domains and *Aureococcus anophagefferens* shares this homology in part. The other three algae of group 2 share a specific homology as well. The aureochrome designations consist of a species abbreviation (*Phaeodactylum tricornutum* (Pt), *Fragilariopsis cylindrus* CCMP 1102 (Fc), *Pseudo-nitzschia multiseries* (Pm), *Thalassiosira pseudonana* (Tp), *Thalassiosira oceanica* (To), *Ectocarpus siliculosus* (Es), *Fucus distichus* subsp. Evanescens (Fd), *Aureococcus anophagefferens* (Aa), *Chattonella marina* var. Antiqua (Cm), *Ochromonas danica* (Od), *Nannochloropsis gaditana* CCMP526 (Ng), *Vaucheria frigida* (Vf)) and either the NCBI (http://www.ncbi.nlm.nih.gov/) gene accesion number (three letters and numbers) or the JGI (http://www.jgi.doe.gov/) protein ID (only numbers).
